# Supplementary material for: An integrated multi-omics approach identifies the landscape of interferon-α-mediated responses of human pancreatic beta cells
Source: Nat Commun. 2020 May 22;11:2584. doi: 10.1038/s41467-020-16327-0 (PMC7244579; doi:10.1038/s41467-020-16327-0)
Supplement: Supplementary file 1 — Supplementary Information [file 41467_2020_16327_MOESM1_ESM.pdf]

An integrated multi-omics approach identifies the landscape of interferon-α-mediated responses of human pancreatic beta cells

Colli at al.

Supplementary Figures

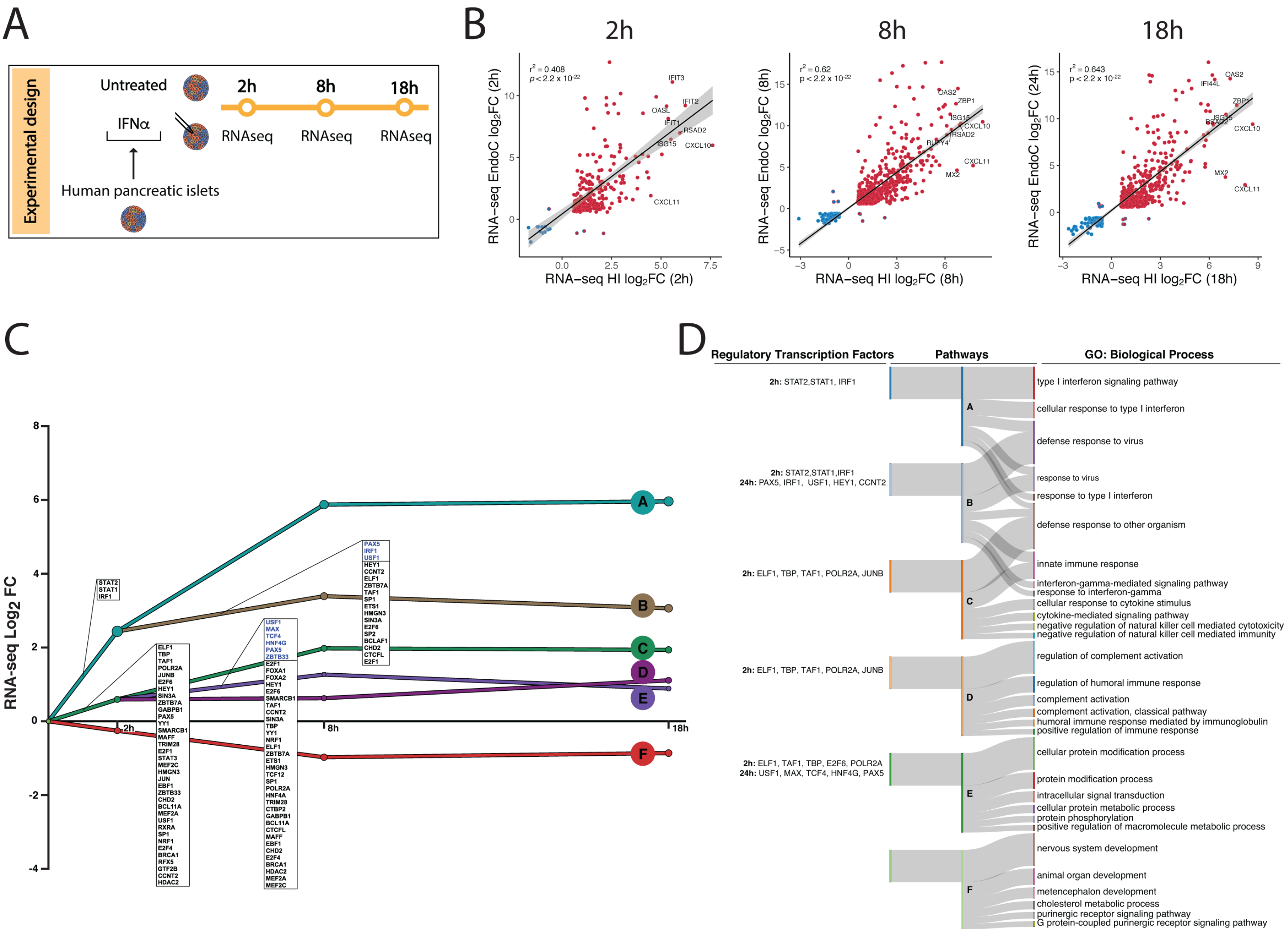

Supplementary Figure 1. Characterization of the global transcriptional responses of human islets to interferon-α. A. Schematic representation of the present experimental design. In brief, pancreatic human islets were exposed or not to IFNα (2000 U/ml) for the indicated time points and RNA-sequencing was performed (n = 6). **B.** Correlation between RNA-seq of EndoC-βH1 cells (n = 5) and RNA-seq of human islets (n = 6) exposed to INFα. The x axis represents the log_2FC of DEGs from human islets RNA-seq and the y axis the DEG from EndoC-βH1 cells RNA-seq at different time points. The up-regulated ($log_2FC > 0.58$, $FDR < 0.05$) and down-regulated ($log_2FC < -0.58$, $FDR < 0.05$) mRNAs in human islets are filled in red and blue, respectively. The up-regulated ($log_2FC > 0.58$, $FDR < 0.15$) or down-regulated ($log_2FC < -0.58$, $FDR < 0.15$) mRNAs in EndoC-βH1 cells are represented by red and blue borders, respectively. **C.** The DREM[1] regulatory paths summarizing the temporal patterns of the differentially expressed genes (DEG) detected by RNA-seq ($|log_2FC| > 0.58$ and $FDR < 0.05$, n = 6). The x axis represents the time and the y axis the mRNA log_2FC. Each path corresponds to a set of co-expressed genes. Split nodes (circles) represent a temporal event where a group of genes co-expressed up to that point diverge in expression,

most probably due to regulatory events. These annotations are placed on the path immediately after the split to indicate whether the transcription factor (TF) controls the upper or lower paths following the split. In blue are the TFs upregulated at the respective time points of the RNA-seq. **D.** Transcription factors and biological processes (GO) enriched in the main pathways defined by the DREM model (FDR < 0.05).

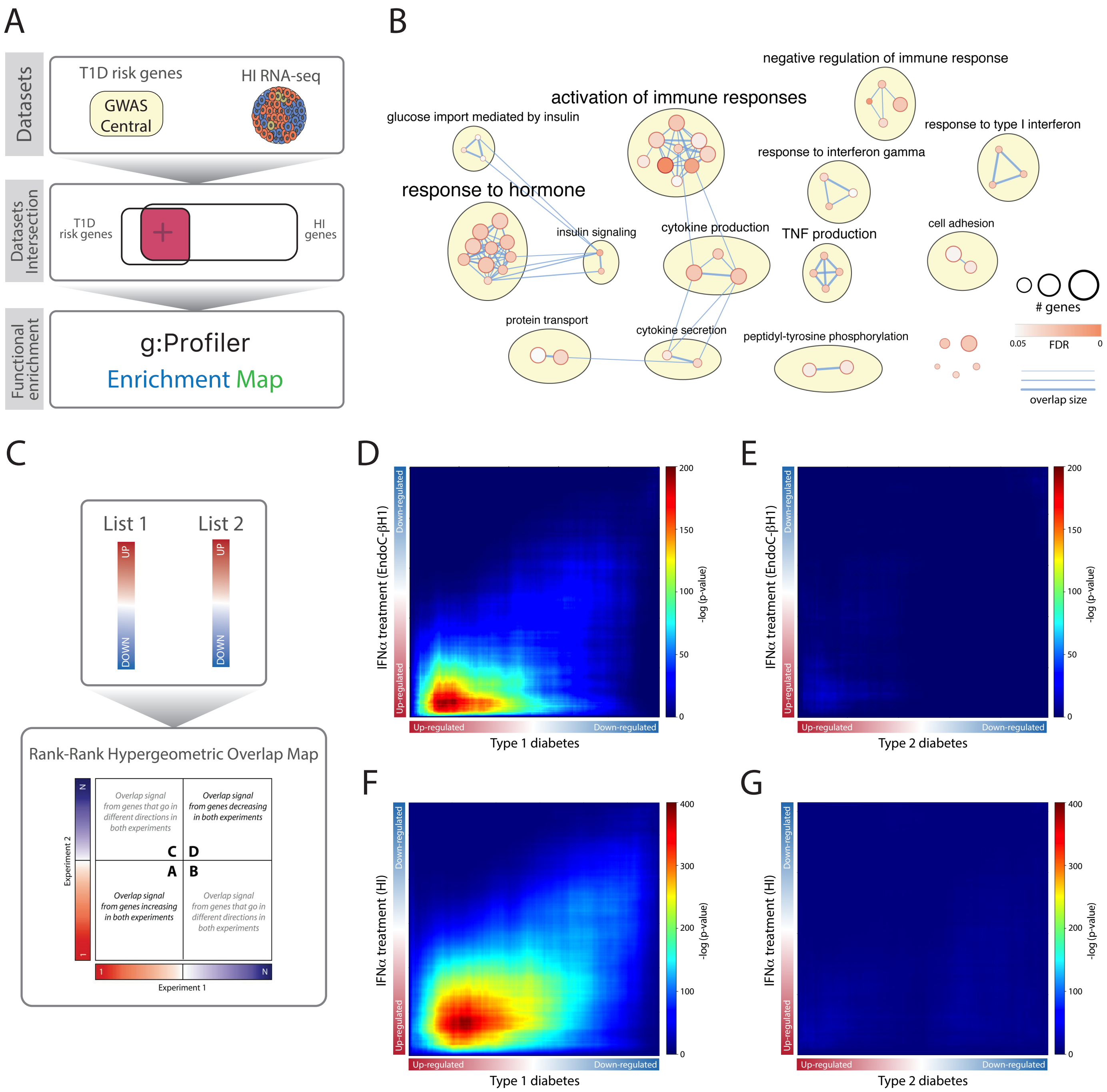

Supplementary Figure 2. Type I interferon-induced genes are enriched among T1D candidate genes and the *in vitro* modelling of this cytokine partially recapitulates the beta cell gene expression signature from T1D individuals. . A. Summary of the approach used to evaluate the biological processes regulated by T1D candidate genes expressed in human islets. Initially, two lists were generated, one containing the T1D risk genes (see Methods) and another the genes expressed in pancreatic human islets (RPKM > 0.5, (GSE108413[2]). Next, these two lists were intersected and functional enrichment analysis was performed using the overlapping genes. **B.** Enrichment map of the biological processes (GO) significantly overrepresented in T1D candidate genes expressed in pancreatic human islets performed as described in Methods. **C.** Schematic illustration of the steps followed for the Rank-Rank Hypergeometric Overlap (RRHO) analysis. In brief, ranked-lists of genes based on the log_2FC (T1D or T2D

vs non-diabetic individuals) were generated (List 1) and compared to similarly ranked lists of EndoC-βH1 cells (**D and E**) and human islets (**F and G**) exposed to IFNα for 24 or 18h, respectively, vs untreated cells (List2). Next, the RRHO map was used to screen the sample permutation maps for the higher overlap in genes among the lists. **D, E, F and G.** RRHO map comparing the gene expression profile of EndoC-βH1 cells (**D**) or human islets (HI) (**F**) treated with IFNα to the one present in primary beta cells of individuals affected by T1D[3] identified by RNA-seq. This IFNα-induced profile of EndoC-βH1 cells (**E**) and human islets (HI) (**G**) was also compared against the mRNA expression profile of beta cells isolated from individuals affected by T2D[4], to exclude the impact of metabolically-induced changes.

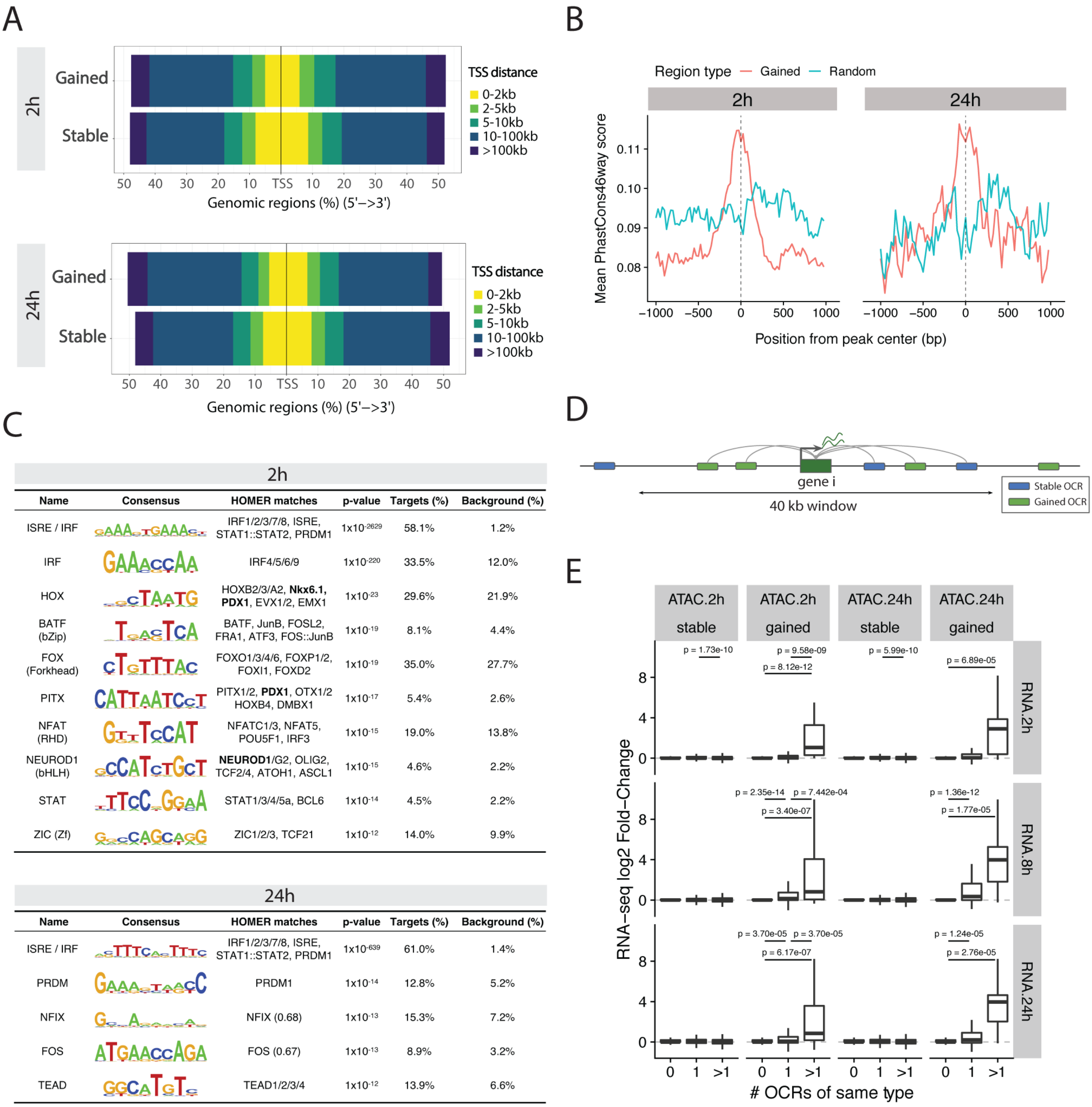

2h

Name	Consensus	HOMER matches	p-value	Targets (%)	Background (%)
ISRE / IRF	GAAAGTGAAAGT	IRF1/2/3/7/8, ISRE, STAT1::STAT2, PRDM1	$1x10^{-2629}$	58.1%	1.2%
IRF	GAAACCAA	IRF4/5/6/9	$1x10^{-220}$	33.5%	12.0%
HOX	GCTAATG	HOXB2/3/A2, **Nkx6.1, PDX1**, EVX1/2, EMX1	$1x10^{-23}$	29.6%	21.9%
BATF (bZip)	TGACTCA	BATF, JunB, FOSL2, FRA1, ATF3, FOS::JunB	$1x10^{-19}$	8.1%	4.4%
FOX (Forkhead)	CTGTTTAC	FOXO1/3/4/6, FOXP1/2, FOXI1, FOXD2	$1x10^{-19}$	35.0%	27.7%
PITX	CATTAATCCT	PITX1/2, **PDX1**, OTX1/2 HOXB4, DMBX1	$1x10^{-17}$	5.4%	2.6%
NFAT (RHD)	GTTTCCAT	NFATC1/3, NFAT5, POU5F1, IRF3	$1x10^{-15}$	19.0%	13.8%
NEUROD1 (bHLH)	GCCATCTGCT	**NEUROD1**/G2, OLIG2, TCF2/4, ATOH1, ASCL1	$1x10^{-15}$	4.6%	2.2%
STAT	TTCCGGAA	STAT1/3/4/5a, BCL6	$1x10^{-14}$	4.5%	2.2%
ZIC (Zf)	GCCCAGCAGG	ZIC1/2/3, TCF21	$1x10^{-12}$	14.0%	9.9%

24h

Name	Consensus	HOMER matches	p-value	Targets (%)	Background (%)
ISRE / IRF	ACTTTCACTTTC	IRF1/2/3/7/8, ISRE, STAT1::STAT2, PRDM1	$1x10^{-639}$	61.0%	1.4%
PRDM	GAAAGTAACC	PRDM1	$1x10^{-14}$	12.8%	5.2%
NFIX	GCAAGAACAG	NFIX (0.68)	$1x10^{-13}$	15.3%	7.2%
FOS	ATGAACCAGA	FOS (0.67)	$1x10^{-13}$	8.9%	3.2%
TEAD	GGCATGTC	TEAD1/2/3/4	$1x10^{-12}$	13.9%	6.6%

Supplementary Figure 3. Gained open chromatin regions are mainly localized distally to gene transcription starting sites (TSSs), evolutionary conserved and enriched in transcription factors (TFs) binding motifs. A. Distribution of the open chromatin regions (OCRs) distance to the closest gene TSS in different classes and time points. **B.** Mean sequence conservation score of gained open chromatin regions (2 and 24h) and randomized sets in placental mammals. Peaks are extended from the center to 1kb in each direction and mean score was calculated in 50bp windows. **C.** TFs binding motifs enriched in regions of IFNα-induced gained open chromatin. **D.** Scheme representing the strategy to analyze OCRs and their potential target genes. (n = 4, unadjusted p-values were obtained using the hypergeometric test from the HOMER package[5]). **E.** mRNA log_2 fold-changes detected by RNA-seq are related to the type

and the number of REs. Boxplot limits show upper and lower quartiles, centered on the median value. Whiskers extend to 1.5 times the interquartile range (n = 5, p-values were adjusted using the Benjamini-Hochberg method, two-sided Mann-Whitney test).

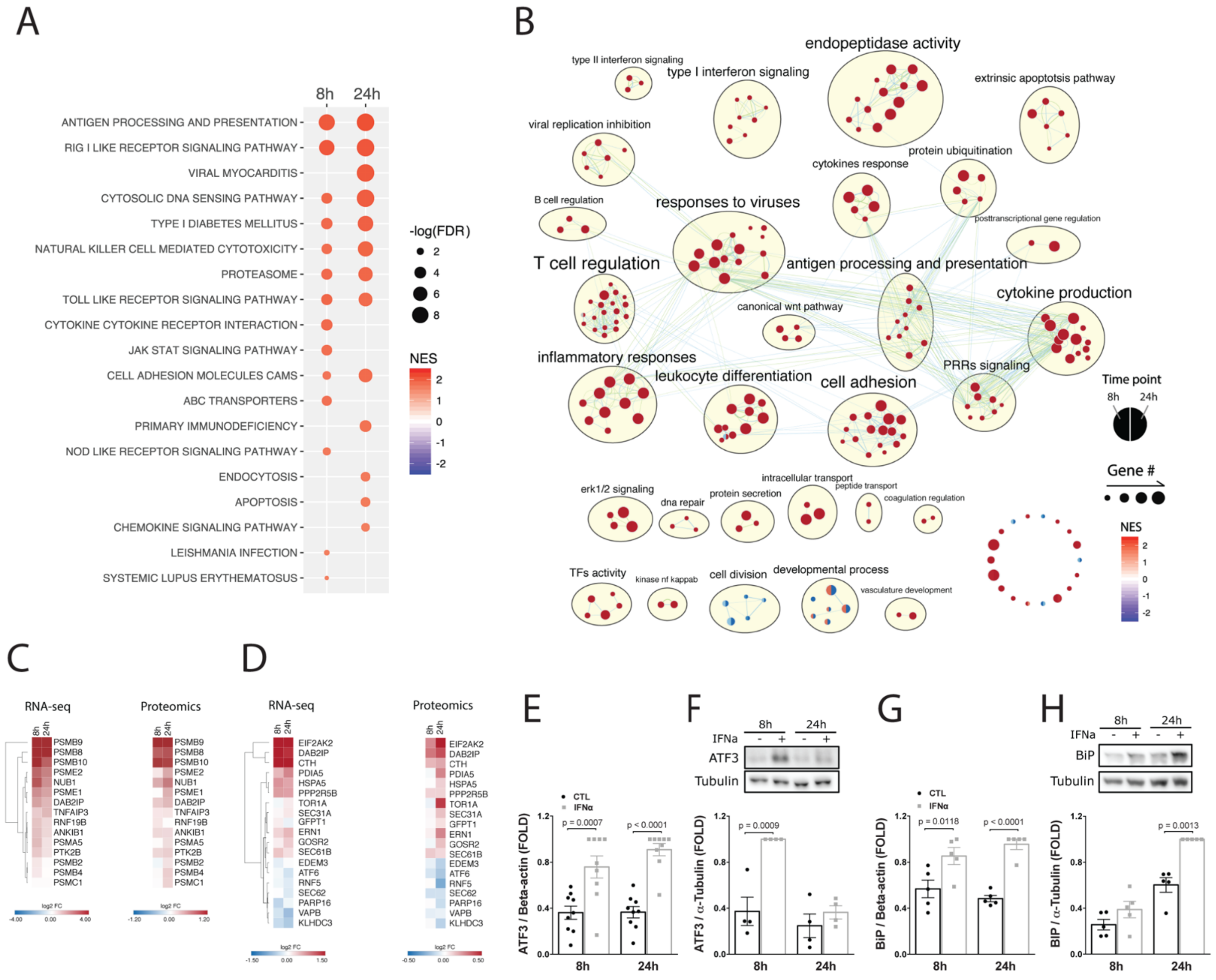

Supplementary Figure 4. IFNα decreases expression of proteins via proteasome activation and triggering of ER stress in EndoC-βH1 cells . A, B. Gene set enrichment analysis (GSEA) of KEGG pathways (**A**) and enrichment map of biological processes (Gene Ontology) (**B**) significantly enriched among proteins modified by IFNα in EndoC-βH1 cells. **C, D.** mRNAs and proteins involved in proteasomal degradation and triggering ER stress are significantly modified by IFNα in EndoC-βH1 cells (**C:** (n = 5), **D**: (n = 4), genes/proteins were selected if they present a FDR < 0.05 in at least one time point/condition). **E to H.** Validation of key mRNAs and proteins involved in protein folding and degradation. EndoC-βH1 cells were treated or not with IFNα for the indicated time points in independent experiments and expression of the stress-responsive transcription factor *ATF3* (**E, F**) and the chaperone BiP (*HSPA5*) (**G, H**) were evaluated at the mRNA (**E, G**) and protein levels (**F, H**). (for **E** (n = 9), **F** (n = 4), **G** and **H** (n = 5), mean ± SEM, ANOVA with Bonferroni correction for multiple comparisons). Source data are provided as a Source Data file.

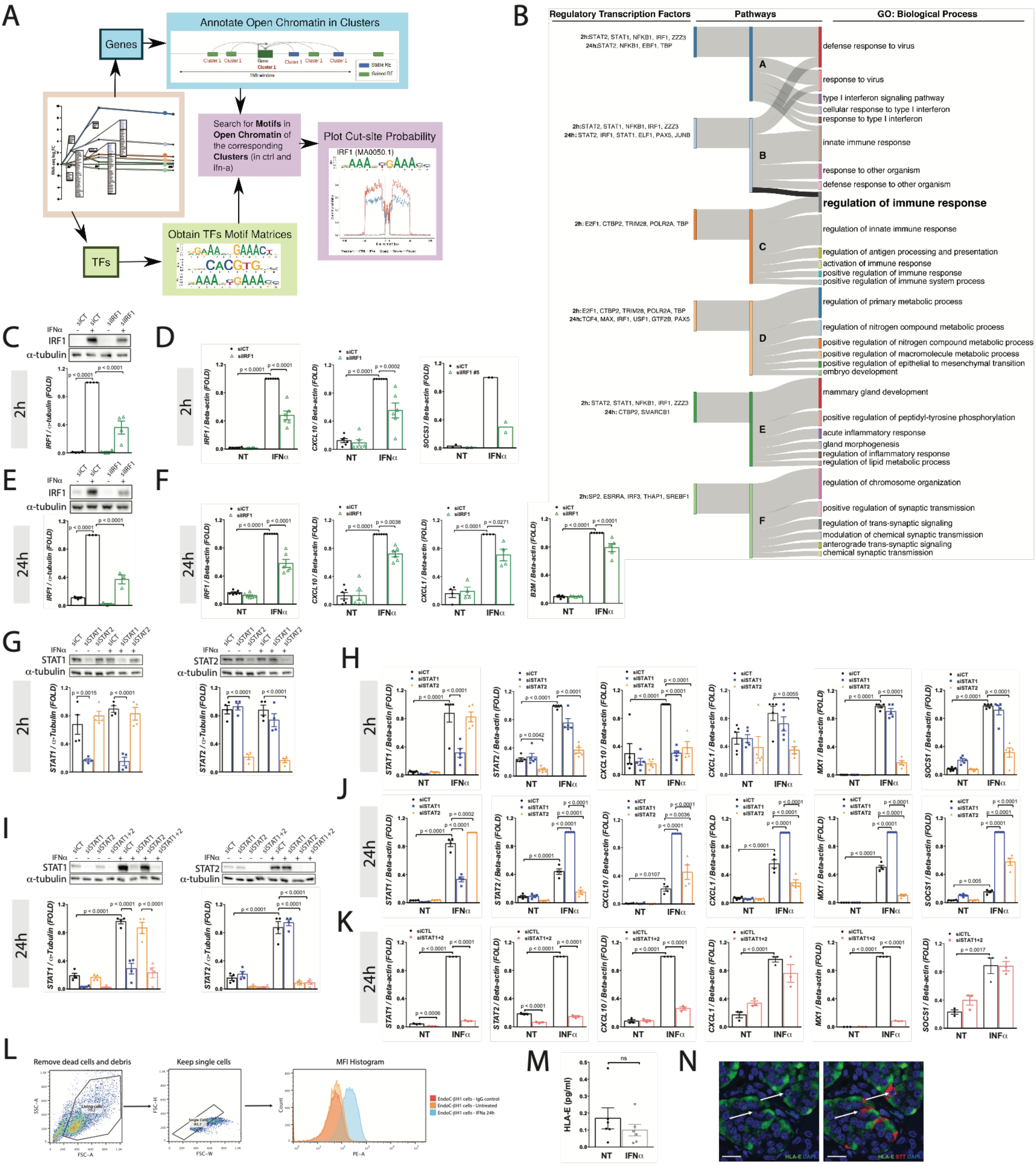

Supplementary Figure 5. Identification of key transcription factors regulating EndoC-βH1 cells responses to IFNα. A. Flowchart of the experimental approach: First, the DREM model divided the differentially expressed genes in pathways based on their expression pattern and pointed out TFs potentially controlling paths diverging over time. Second, the coordinates of the genes classified in each

of the pathways were obtained. Third, OCRs at 2 and 24h were associated to the nearest gene classified in one of the paths at 2 and 24h, respectively. Forth, motif matrices of the putative TFs regulating a specific path were identified. Fifth, matches for these matrices were searched for in the respective subset of OCRs annotated to the cluster regulated by the respective TFs. Sixth, the difference in cutting probability between control and IFNα samples was plotted. **B.** TFs and biological processes (GO) enriched in the main pathways defined by the DREM model (FDR < 0.05). **C to K.** EndoC-βH1 cells were transfected with a control non-specific siRNA (siCT) or previously validated siRNAs[6,7] against *IRF1* (siIRF1) (**C to F**), *STAT1* (siSTAT1) (**G to J**), *STAT2* (siSTAT2) (**G to J**) or *STAT1* plus *STAT2* (siSTAT1+2) (**I and K**). After 48h of recovery, the knockdown efficiency was confirmed at the protein level by Western blot and quantified by densitometry **(C, E, G, I**). The values were normalized by the housekeeping protein α-tubulin and then by the highest value of each experiment considered as 1. The Western blot images are representative of three (**E**) and four (**C, G, I**) independent experiments (ANOVA with Bonferroni correction for multiple comparisons). The mRNA induction of key IFNα-target genes (**D, F, H, J and K**) was determined by real-time PCR at the indicated time points. The values were normalized by the housekeeping gene *β-actin* and then by the highest value of each experiment considered as 1 (for **D:** IRF1 and CXCL10 (n = 6), SOCS1 (n = 2); for **F**: IRF1 and CXCL10 (n = 6), CXCL1 (n = 4); B2M (n = 5); for **H**: all (n = 5); for **J**: all (n = 4); for **K**: all (n = 3), ANOVA with Bonferroni correction for multiple comparisons). **L.** Gating strategy used to evaluate HLA-E expression on EndoC-βH1 cells exposed or not to IFNα for 24h (see Supplementary Methods, the same gating strategy was used for MHC Class I analysis). **M.** HLA-E protein in the supernatant of EndoC-βH1 cells exposed or not to IFNα for 24h was measured by ELISA. (n = 6, ns: non-significant, two-sided paired *t* test) **N.** Higher magnification image of an islet from a T1D donor demonstrating that HLA-E (green) does not localize to delta cells (somatostatin; red – white arrows). DAPI – dark blue. Scale bar 20 µm. Source data are provided as a Source Data file.

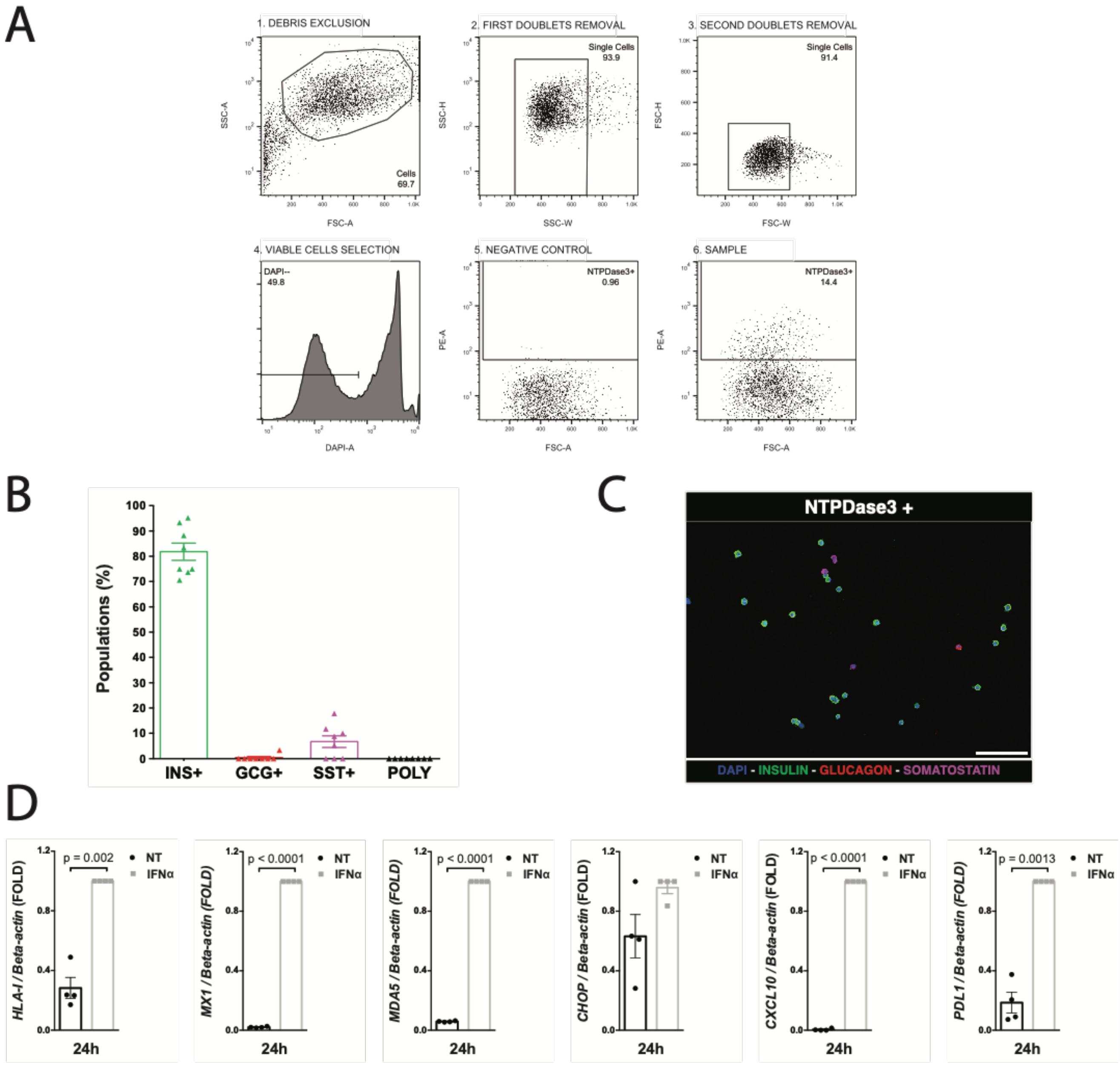

Supplementary Figure 6. Evaluation of interferon-α-induced genes on FACS-purified primary human beta cells. Pancreatic human islets were FACS-sorted using a beta cell specific cell surface marker (*NTPDase3*) (see Methods). **A.** Gating strategy used to obtain purified human beta cells. **B.** The islet cell population frequency in the beta cell enriched fraction was determined by immunocytochemistry (ICC) with antibodies against insulin (INS), glucagon (GCG) or somatostatin (SST) (8 fields were analysed from 2 independent experiments). **C.** Representative ICC image of the beta cell enriched fraction after FACS-sorting of pancreatic human islets. Image representative of 2 independent experiments. Scale bar 100 μm. **D.** mRNA expression of *HLA class I (ABC), MX1, MDA5, CHOP, CXCL10* and *PDL1* was evaluated by real-time RT-PCR on FACS-purified human beta cells exposed or not to IFNα for 24h. The values were normalized by the housekeeping gene *β-actin* and then by the highest value of each experiment considered as 1 (n = 4, two-sided paired *t* test). Source data are provided as a Source Data file.

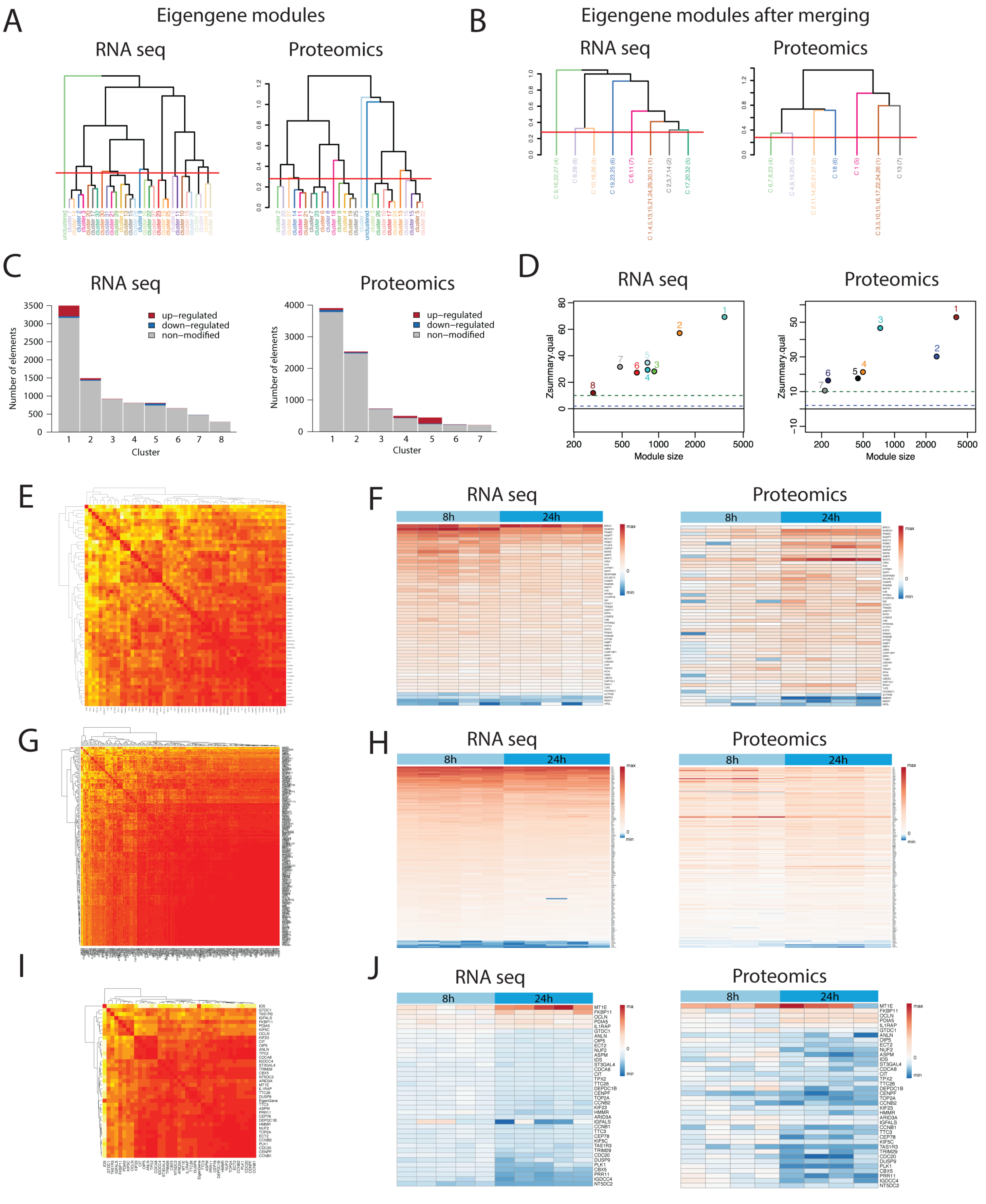

Supplementary Figure 7. Description of interferon-α-regulated modules of genes and proteins. A. Dendrogram of consensus module eigengenes identified by WGCNA mRNA and protein clustering. **B.**

Modules obtained after merging eigengenes groups whose expression profiles were similar. A threshold of eigengene correlation of 0.75 (corresponding to the dissimilarity threshold 0.25) was used for the merging. **C.** Number and status of the mRNAs and proteins in each module from RNA-seq and proteomics of EndoC-βH1 cells. **D.** Module quality is represented as the Zsummary for each module from the resulting networks from RNA-seq and proteomics of EndoC-βH1 cells. For this analysis, the original dataset was re-sampled 1000-times to create reference and test sets and then module quality was evaluated using density and separability metrics and represented as the Zsummary for each module. Zsummary > 2 indicates moderate and Z > 10 high quality/robustness for each module[8]. **E, G, I.** Heatmap plots depicting the relation between genes and proteins in each module. Progressively darker colors correspond to higher correlations. **F, H, J.** Log_2 fold-changes of mRNAs and proteins present in modules #1 (**F**), #2 (**H**) and #5 (**J**), which were generated as described before in Figure 4B and C. The present results show RNA-seq (n = 5) and proteomics (n = 4) data of EndoC-βH1 cells.

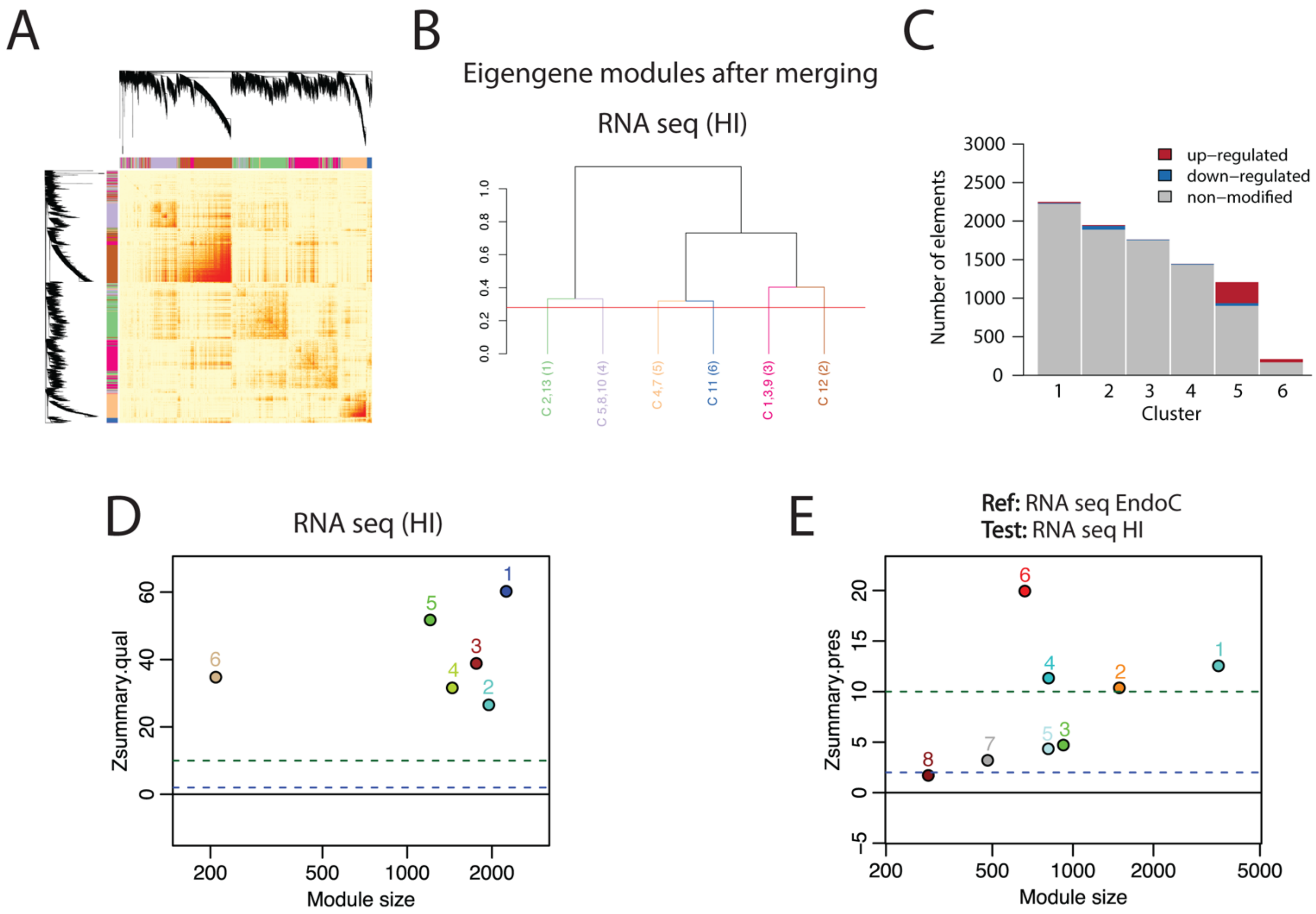

Supplementary Figure 8. Weighted correlation network analysis (WGCNA) of human pancreatic islets RNA-seq. A. Heatmap representation of the topological overlap matrix. Rows and columns correspond to single genes, light colors represent low topological overlap, and progressively darker colors represent higher topological overlap. The corresponding gene dendrograms and initial module assignment are also displayed. **B.** Modules obtained after merging eigengenes groups whose expression profiles were similar. A threshold of eigengene correlation of 0.75 (corresponding to the dissimilarity threshold 0.25) was used for the merging. **C.** Number and status of the mRNAs in each module based on the RNA-seq data from pancreatic human islets. **D.** Module quality is represented as the Zsummary for each module from the resulting networks. For this analysis, the original dataset was re-sampled 1000-times to create reference and test sets and then module quality was evaluated using density and separability metrics, and represented as the Zsummary for each module. Zsummary > 2 indicates moderate and Z > 10 high quality/robustness for each module[8]. **E.** Similarity between EndoC-βH1 cells mRNAs modules (reference set) and the human islets RNA-seq modules (test set) was evaluated as described in (**C**), but using density and intramodular connectivity metrics. Module preservation is represented as the Zsummary for each module. Zsummary > 2 indicates moderate and Z > 10 high preservation for each module[8]. The present results show RNA-seq data (n = 6) of pancreatic human islets.

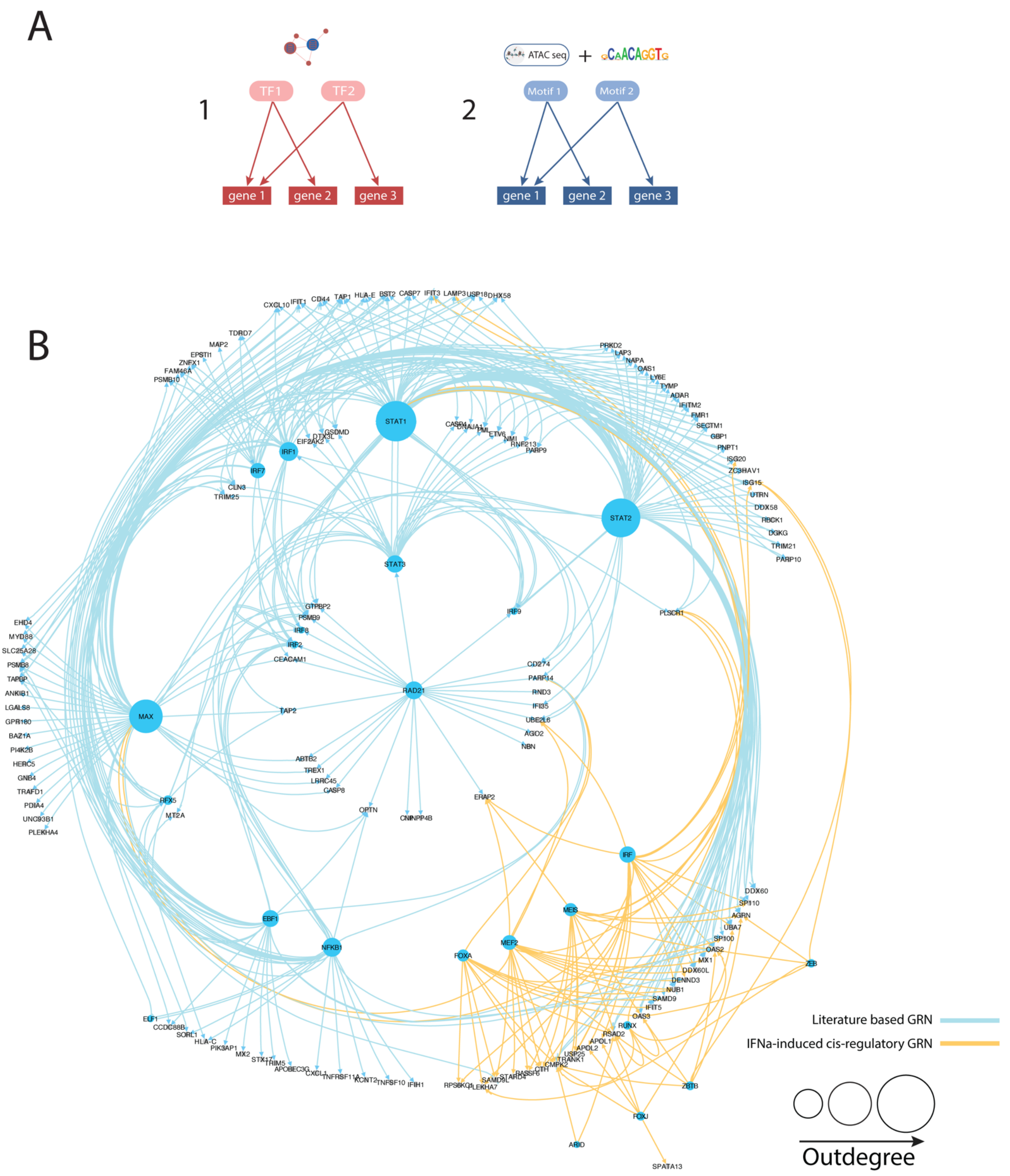

Supplementary Figure 9. Reconstructing the gene regulatory networks induced by interferon-α in EndoC-βH1 cells. A. A gene regulatory network of module #2 was built combining data from two sources: 1) Transcription factor (TF)-target information obtained from publicly available data-repository of regulations (www.regnetworkweb.org). Enriched regulators (FDR < 0.01 and minimum number of

connections = 4) were identified and added to the network if they were not already present. 2) Previously recognized *de novo* HOMER motifs were linked to their target genes using *annotatePeaks.pl.* **B.** Gene regulatory network from module #2. Light blue edges represent the literature-based network. Orange edges indicate the cis-regulatory network presently identified. The size of the nodes is proportional to the outdegree of the regulatory components. The present results were based on RNA-seq (n = 5) and proteomics (n = 4) data of EndoC-βH1 cells.

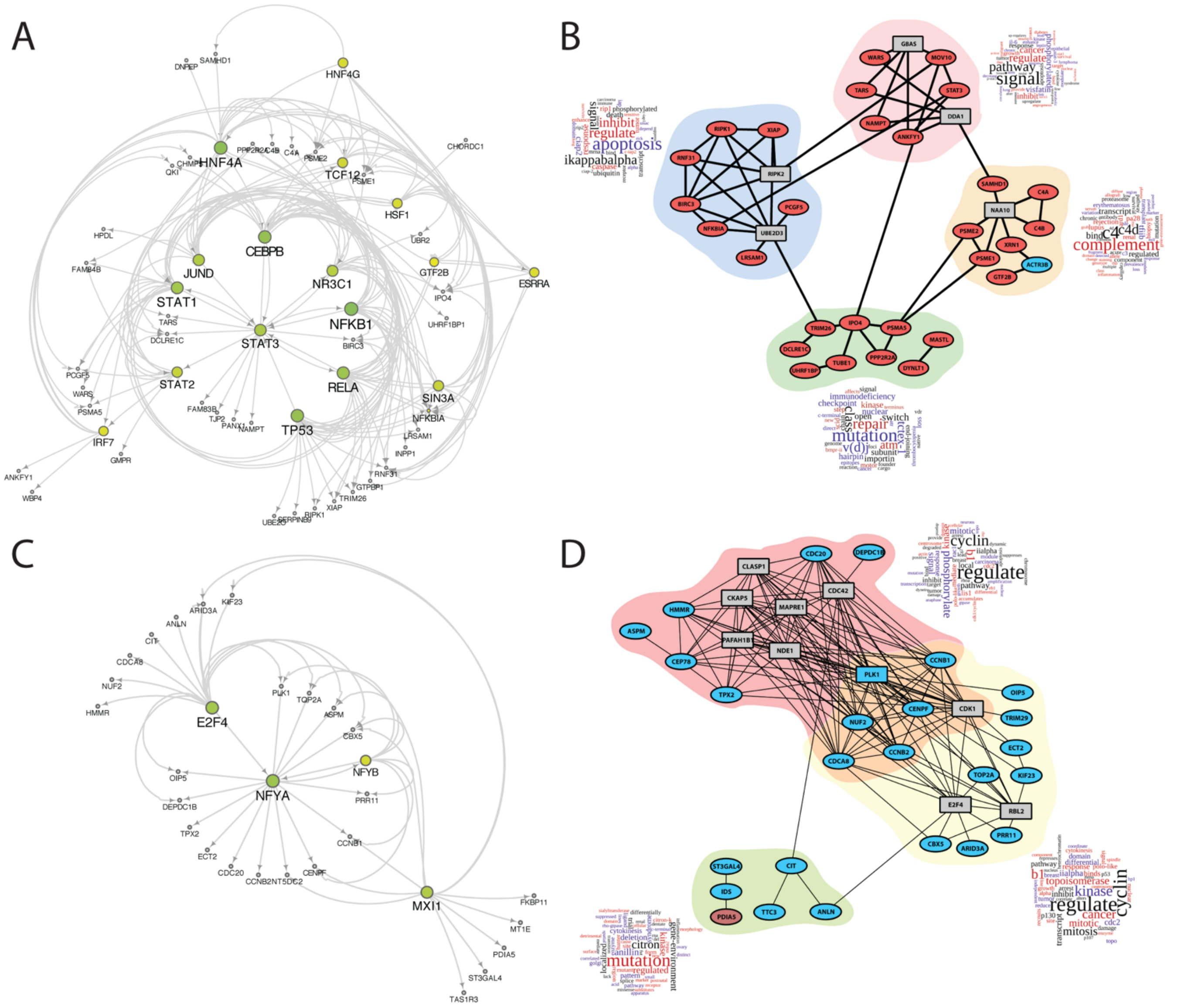

Supplementary Figure 10. Gene regulatory networks and protein-protein interaction (PPI) networks from modules #1 and #5. A, C. Gene regulatory networks of modules #1 (**A**) and #5 (**C**) were built using TF-target information obtained from publicly available data-repository of regulations (www.regnetworkweb.org). Enriched regulators (FDR < 0.05 and minimum number of connections = 5) were identified and added to the network if they were not already present. The size of the TFs nodes is proportional to the outdegree. **B, D.** The PPI network of modules #1 (**B**) and #5 (**D**) was done using the InWeb InBioMap database[9]. Enriched proteins (FDR < 0.05 and minimum number of connections = 5, represented as squares) were identified and added to the network if they were not already present. Red fill identifies upregulated proteins, blue fill indicates downregulated proteins and gray fill equal-regulated. Colored regions delimitate communities of proteins identified using the EAGLE algorithm, as described in Material and Methods. The wordcloud next to each community presents their enriched geneRIFs terms. The present results were based on RNA-seq (n = 5) and proteomics (n = 4) data of EndoC-βH1 cells.

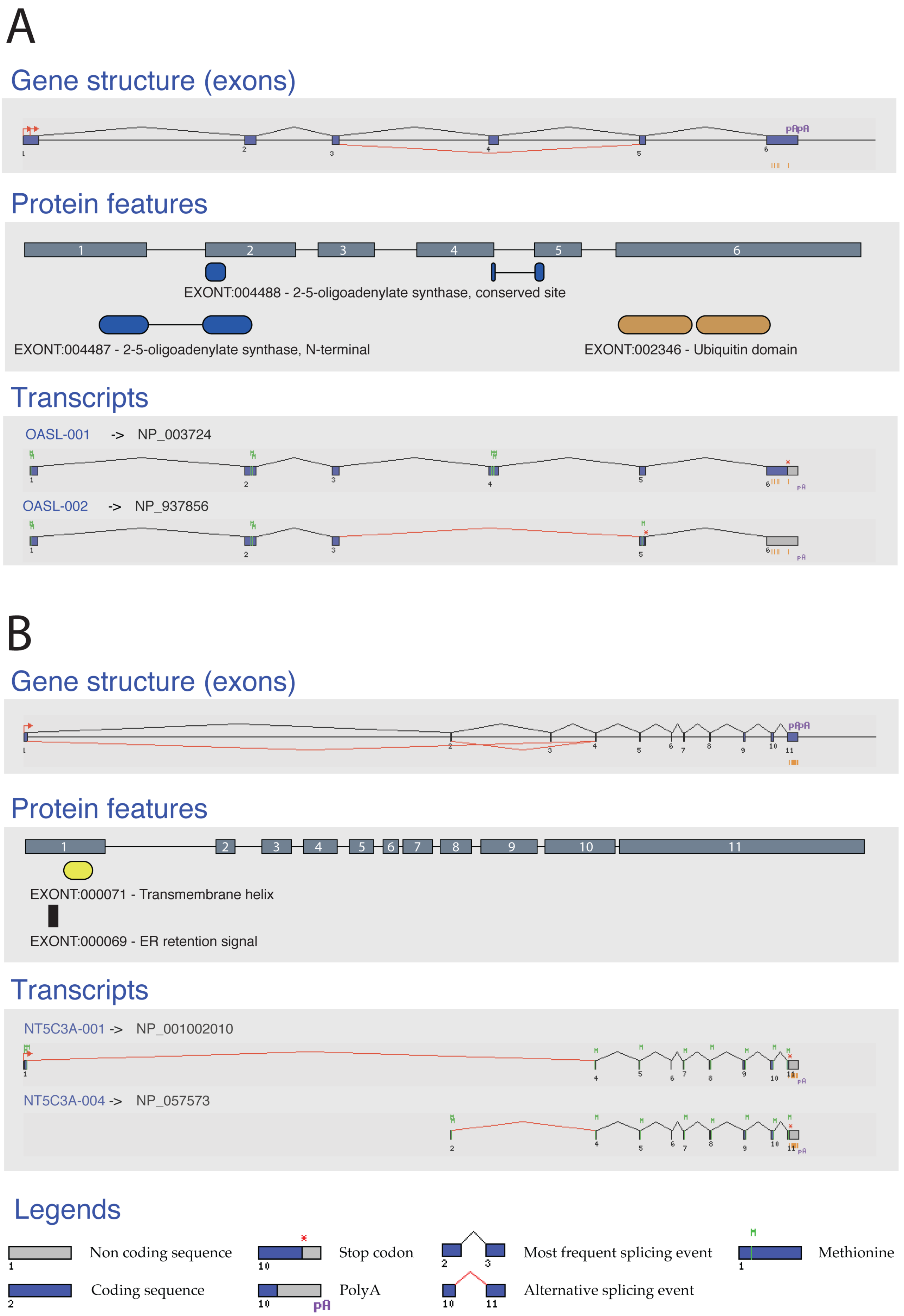

Supplementary Figure 11. Interferon-α leads to alternative exon usage with functional impact. Graphical representation of *OASL* (**A**) and *NT5C3A* (**B**) gene structure with all its exons and the most

frequent alternative exon skipping/retention events (red). In the middle it is shown the protein features associated to their respective exons and at the bottom the transcripts regulated by IFNα that are affected by the usage of alternative exons.

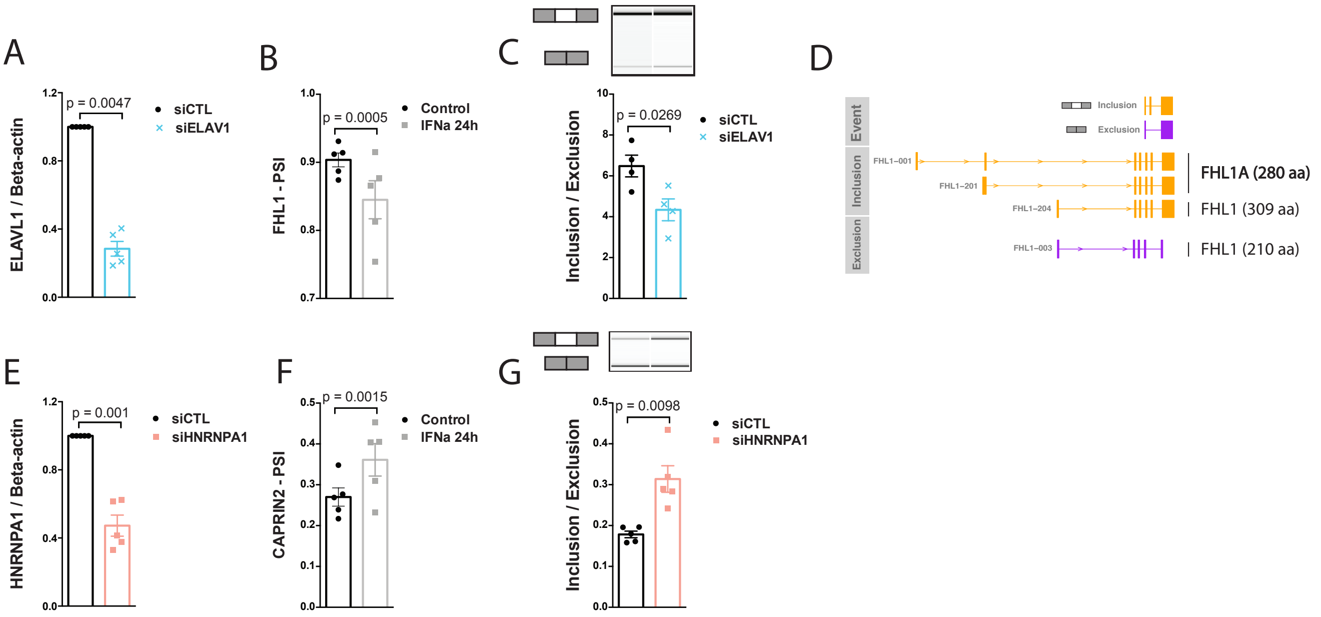

Supplementary Figure 12. Inhibition of ELAVL1 and HNRNPA1 reproduces IFNα-induced changes in alternative splicing. A and E. ELAVL1 (**A**) and HNRNPA1 (**E**) were silenced in EndoC-βH1 cells using specific siRNAs and their mRNA expression were evaluated by real-time RT-PCR. The values were normalized by the housekeeping gene β-actin and then by the highest value of each experiment considered as 1 (for **A** and **E** (n = 5), mean ± SEM, two-sided paired *t* test). **B and F**. *FHL1* and *CAPRIN2* percent splice-in (PSI) values in RNA-seq of EndoC-βH1 cells exposed to IFNα for 24h. PSI represents the ratio of normalized read counts, indicating inclusion of the transcript element over the total normalized reads for that event (inclusion and exclusion reads) (for **B** and **F** (n = 5), mean ± SEM, adjusted p-values (FDR). **C and G.** Confirmation of exon 6 (*FHL1*) (**C**) and exon 5 (*CAPRIN2*) (**G**) inclusion in EndoC-βH1 cells after silencing of *ELAVL1* and *HNRNPA1*, respectively. cDNA was amplified by RT-PCR using primers located in the up-stream and down-stream exons of the splicing event. The PCR products were analysed by automated electrophoresis using a Bioanalyzer 2100 machine and quantified by comparison with a loading control. (for **C** (n = 4) and **G** (n = 5), mean ± SEM, two-sided paired *t* test). **D.** Schematic representation of the transcripts expressed in EndoC-βH1 cells with differential usage of exon 6 on *FHL1* and the proteins encoded by these transcripts (aa: amino acids). Source data are provided as a Source Data file.

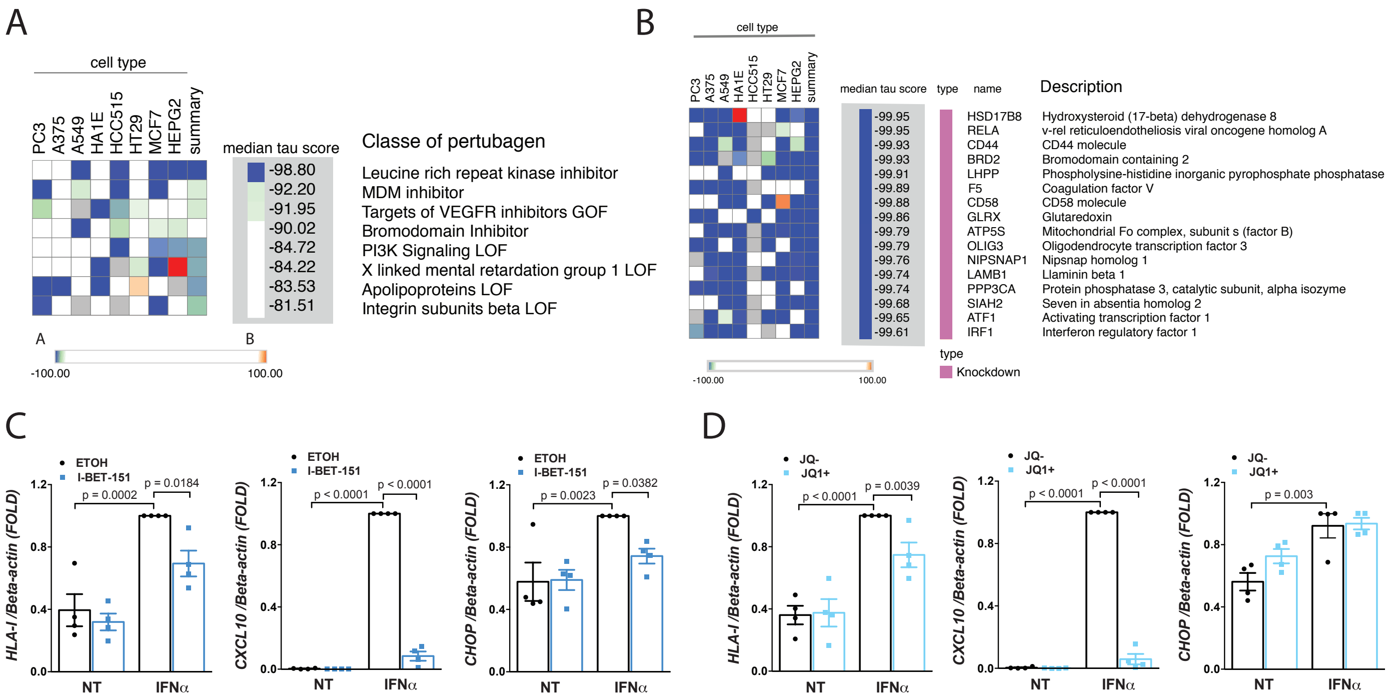

Supplementary Figure 13. Bromodomain inhibitors decrease the type I interferon-induced signature in pancreatic human islets. A. Classes of drugs that have an opposite signature to the one shared among beta cells of individuals with T1D and human pancreatic islets exposed to IFNα for 18h (see Supplementary Fig. 2D). **B.** Knockdown of the gene *BRD2* (bromodomain containing 2) generates an inversely related signature in comparison to IFNα in the Connectivity MAP database[10]. **C, D.** Dispersed human islets were pretreated for 2h with the bromodomain inhibitors I-BET-151 (1 μM) (**C**) or JQ1+ (0.4 μM) (**D**) and then exposed to IFNα for 24h. After this period the cells were collected and the mRNA expression of *HLA class I (ABC)*, the chemokine *CXCL10* and the ER stress marker *CHOP* were evaluated by real-time RT-PCR. The vehicle Ethanol and an inactive enantiomer (JQ1-) were used as respective controls for I-BET-151 and JQ1+. (for **C** and **D** (n = 4) mean ± SEM, ANOVA with Bonferroni correction for multiple comparison). Source data are provided as a Source Data file.

Supplementary Tables

Supplementary Table 1. List of *FMR1* targets for which mRNA binding and protein regulation have been reported in more than one study[11].

Gene name	Gene symbol
Apoptosis associated tyrosine kinase	*AATK*
Aldolase, fructose-bisphosphate A	*ALDOA*
Adaptor related protein complex 2 subunit beta 1	*AP2B1*
APC, WNT signaling pathway regulator	*APC*
Amyloid beta precursor protein	*APP*
Activity regulated cytoskeleton associated protein	*ARC*
Rho guanine nucleotide exchange factor 12	*ARHGEF12*
Calcium/calmodulin dependent protein kinase II alpha	*CAMK2A*
Catenin beta 1	*CTNNB1*
Dystroglycan 1	*DAG1*
DSCS large MAGUK scaffold protein 4	*DLG4*
DLG associated protein 4	*DLGAP4*
Eukaryotic translation elongation factor 2	*EEF2*
Fragile X mental retardation 1	*FMR1*
FUS RNA binding protein	*FUS*
Gamma-aminobutyric acid type A receptor beta1 subunit	*GABRB1*
Gamma-aminobutyric acid type A receptor delta subunit	*GABRD*
Heterogeneous nuclear ribonucleoprotein A2/B1	*HNRNPA2B1*
Potassium voltage-gated channel subfamily C member 1	*KCNC1*
Potassium voltage-gated channel subfamily D member 2	*KCND2*
Microtubule associated protein 1B	*MAP1B*
Microtubule associated protein 2	*MAP2*
Myelin basic protein	*MBP*
Matrix metallopeptidase 9	*MMP9*
Neuroligin 2	*NLGN2*
Nuclear receptor subfamily 3 group C member 1	*NR3C1*
Oligophrenin 1	*OPHN1*
Protocadherin 10	*PCDH10*
Piccolo presynaptic cytomatrix protein	*PCLO*
Phosphatidylinositol-4,5-bisphosphate 3-kinase catalytic subunit beta	*PIK3CB*
Plakophilin 4	*PKP4*
Proteolipid protein 1	*PLP1*
Protein phosphatase 2 catalytic subunit alpha	*PPP2CA*
Protein tyrosine phosphatase, non-receptor type 5	*PTPN5*
Rac family small GTPase 1	*RAC1*
Regulator of G protein signaling 5	*RGS5*
Ras homolog family member A	*RHOA*
Superoxide dismutase 1	*SOD1*
Spen family transcriptional repressor	*SPEN*
Voltage dependent anion channel 1	*VDAC1*

Supplementary Table 2. Characteristics of the human islets and tissue donors.

Islets donors									
	Age (y)	Gender	BMI (kg/m^2)	Cause of death	Beta cell purity (%)	ICU stay (days)	Mean glycemia (mg/dl)	Dithizone (%)	Cold ischemia time (hours)
Donor 1	63	F	27.3	Stroke	58	4	110	80	15
Donor 2	74	M	26	Cerebral hemorrhage	46	1	171	90	13
Donor 3	71	F	31.2	Cerebral hemorrhage	46	4	158	80	18
Donor 4	73	M	24.1	Cerebral hemorrhage	NA	12	80	60	15
Donor 5	72	F	22.9	CVD	45	2	156	85	18
Donor 6	56	F	26.6	Cerebral hemorrhage	71	24	210	80	14
Donor 7	66	F	26.4	Trauma	45	11	149	90	14
Donor 8	64	M	31.02	Cardiovascular disease	NA	1	121	50	18
Donor 9	46	M	29.39	Cardiovascular disease	NA	2	169	70	19
Donor 10	79	F	20	Trauma	50	1	59	80	40
Donor 11	63	F	26	Cerebral hemorrhage	45	11	99	50	36
Donor 12	58	F	25.8	Postanoxic encephalopathy	46	1	196	70	17
Donor 13	71	M	27.7	Cerebral hemorrhage	26	3	101	80	14
Donor 14	83	F	28.3	Cardiovascular disease	49	2	134	80	15
**Donor 15***	**67**	**M**	**25.7**	**Trauma**	**48**	**2**	**124**	**50**	**16**
**Donor 16***	**87**	**F**	**23.8**	**Cerebral hemorrhage**	**60**	**4**	**148**	**90**	**14**
**Donor 17***	**67**	**F**	**24.6**	**Cerebral hemorrhage**	**44**	**1**	**147**	**50**	**13**
**Donor 18***	**83**	**F**	**37.1**	**CVD**	**51**	**7**	**117**	**60**	**12**
**Donor 19***	**84**	**F**	**24.5**	**CVD**	**58**	**5**	**124**	**80**	**13**
**Donor 20***	**40**	**F**	**22.5**	**Stroke**	**59**	**1**	**118**	**70**	**14**

*** Samples used for RNA-sequencing**

Tissue donors			
**Identifier**	**Classification**	**Age(y)/Gender**	**Duration of Disease**
21/89	No diabetes	4 / F	
184/90	No diabetes	5 / M	
333/66	No diabetes	16 / M	
146/66	No diabetes	18 / F	
191/67	No diabetes	25 / M	
9310/08	No diabetes	58 / F	
SC115	Type 1 diabetes	16 months / F	3 days
E308	Type 1 diabetes	3 / F	4 weeks
SC41	Type 1 diabetes	4 / F	3 weeks
E261	Type 1 diabetes	18 / F	3 weeks
E560	Type 1 diabetes	41 / F	18 months
DiViD 1	Type 1 diabetes	25 / F	4 weeks
DiViD 3	Type 1 diabetes	34 / F	9 weeks

NA: non-available

ICU: Intensice care unit

CVD: Cardiovascular disease

Supplementary Table 3. List of siRNAs and primers used in the present study.

siRNAs		
	**Distributor**	**Sequence**
**siCT**	Qiagen, Venlo, Netherlands	Allstars Negative Control siRNA, sequence not provided
**Human si*IRF1***	Qiagen, Venlo, Netherlands	5'- CAAGCATGGCTGGGACATCAA -3'
**Human si*STAT1***	Invitrogen, Pasley, UK	5'-GGAUUGAAAGCAUCCUAGAACUCAU-3'
**Human si*STAT2***	Invitrogen, Pasley, UK	5'-CAGCAGCAUGUCUUCUGCUUCCGAU-3'
**Human siELAVL1**	Ambion, Austin, USA	5'-UGUCAAACCGGAUAAACGC-3'
**Human siHNRNPA1**	Ambion, Austin, USA	5'-AUCACUUUUAUAACCAUUC-3'

Primers		
	**Forward**	**Reverse**
	**Sequence (5'-3')**	**Sequence (5'-3')**
**Human *ATF3***	GCTGTCACCACGTGCAGTAT	TTTGTGTTAACGCTGGGAGA
**Human *β-actin***	CTGTACGCCAACACAGTGCT	GCTCAGGAGGAGCAATGATC
**Human *B2M***	TGCTGTCTCCATGTTTGATGTA	GACCAAGATGTTGATGTTGGATAAG
**Human *CHOP***	Qiagen QuantiTect primer, cat# QT00082278	
**Human *CXCL1***	AGAACATCCAAAGTGTGAAC	TTTCTTAACTATGGGGGATG
**Human *CXCL10***	GTGGCATTCAAGGAGTACCTC	GCCTTCGATTCTGGATTCAG
**Human *HLA-E***	TGGTTGCTGCTGTGATATGGA	GCTCCACTCAGCCTTAGAGT
**Human *HLA-I (ABC)***	GAGAACGGGAAGGAGACGC	CATCTCAGGGTGAGGGGCT
**Human *HSPA5***	Qiagen QuantiTect primer, cat# QT00096404	
**Human *IRF1***	CATTCACACAGGCCGATACA	TGGTCTTTCACCTCCTCGATAT
**Human *MX1***	AGACAGGACCATCGGAATCT	GTAACCCTTCTTCAGGTGGAAC
**Human *NT5C3A* -004**	TGTAGCTCGTCCGGTTACCT	ACCAGCTTACAGTTGTCAATGAT
**Human *OASL* (exon 4)**	CCCCTGAGGTCTATGTGAGC	ATCTGTACCCTTCTGCCACG
**Human *PDL1***	CCAGTCACCTCTGAACATGAA	ACTTGATGGTCACTGCTTGT
**Human *RMI2* -004**	CGCCAGACATCTTATGCCCT	TCACAGCAAGGCAGTGTGAA
**Human *SOCS1***	GACGCCTGCGGATTCTAC	GAGGCCATCTTCACGCTAA
**Human *SOCS3***	CCTCGCCACCTACTGAAGGC	CCCGGAGTAGATGTAATA
**Human *STAT1***	GACCCAATCCAGATGTCTATGA	CCCGACTGAGCCTGATTA
**Human *STAT2***	GTTGGCAGTTCTCCTCCTATG	GAAGTCAGCCCAGGACAATAA
**Human ELAVL1**	TTTGATCGTCAACTACCTCCCTC	CTGTGTCCTGCTACTTTATCCCG
**Human HNRNPA1**	ATTTGGACTTTCCCTACCCACTC	CAGCTAGTTTCTATTCCCTGGCA
**Human FHL1**	TTTGCCAAGCATTGCGTGAA	GGCACAGTCGGGACAATACA
**Human CAPRIN2**	TCAAAACTGACCTGCCCTGA	TGGCATTTTTGGGAACTGGG
**Human MDA5**	GAGGAATCAGCACGAGGAATAA	TCAGATGGTGGGCTTTGAC

Supplementary Table 4. List of antibodies and conditions used for the immunofluorescence.

Primary Antibody	Manufacturer and clone	Antigen Retrieval	Antibody Dilution	Incubation time with primary antibody	Secondary Detection System
**Step 1: HLA-E**	Abcam<br>MEM-E/02<br>Mouse Monoclonal<br>Cat#2216	10mM Citrate pH6	1:150	Overnight at 4°C	Goat anti-mouse IgG (H+L) HRP then tyramide Alexa 488 (as per manufacturers protocol; Thermofisher Tyramide SuperBoost kit Cat# B40922)
**Step 2: Glucagon or Somatostatin**	Abcam<br>EP3070<br>Rabbit<br>Monoclonal<br>Cat# 92517	10mM Citrate pH6	1:4000	1h at RT	Goat anti-rabbit IgG (H+L) Alexa Fluor ™-conjugated secondary antibodies (1/400 for 1h)
	Abcam<br>Rat<br>M09204<br>Monoclonal<br>Cat# 30788	10mM Citrate pH6	1:200	1h at RT	Goat anti-rat IgG (H+L) Alexa Fluor ™-conjugated secondary antibodies (1/400 for 1h)
**Step 3: Insulin**	Dako<br>Guinea-Pig Polyclonal<br>Cat#A0546	10mM Citrate pH 6	1:700	1h at RT	Goat anti-guinea-pig IgG (H+L) Alexa Flour™-conjugated secondary antibodies (1/400 for 1h)

Supplementary Table 5. List of antibodies used for Western blot and flow cytometry.

Antibody	Supplier	Identifier	Dilutiuon
HLA-E clone 3D12 (Flow cytometry (FC))	Biolegend	Cat#342602; RRID: AB_1659247	1:250 (FC)
HLA-E clone MEM-E/02 (Western blot (WB) and IF)	Abcam	Cat#ab2216; RRID: AB_302895	1:500 (WB)<br>1:150 (IF)
IRF1	Cell signaling	Cat#8478; RRID: AB_10949108	1:1000 (WB)
phospho-STAT1	Cell signaling	Cat#9167; RRID: AB_561284	1:1000 (WB)
phospho-STAT2	Cell signaling	Cat#88410; RRID:AB_2800123	1:1000 (WB)
total STAT1	Cell signaling	Cat#14495; RRID: AB_2716280	1:1000 (WB)
total STAT2	Cell signaling	Cat#72604; RRID:AB_2799824	1:1000 (WB)
α-tubulin	Sigma	Cat#T9026; RRID:AB_477593	1:5000 (WB)
Peroxidase- conjugated donkey anti-rabbit IgG	Jackson ImmunoResearch	Cat#715-036-152; RRID:AB_2340590	1:1000 (WB)
Peroxidase- conjugated donkey anti-mouse IgG	Jackson ImmunoResearch	Cat#711-036-150; RRID:AB_2340773	1:1000 (WB)
Alexa Fluor 488 goat anti-Guinea-Pig IgG	Life technologies, USA	Cat#A11073; RRID: AB_2534117	1:500 (ICC)
Alexa Fluor 568 rabbit anti-mouse IgG	Life technologies, USA	Cat#A11061; RRID: AB_2534108	1:500 (ICC)
Polyclonal Goat Anti-Mouse Immunoglobulins/RPE Goat	Dako	Cat#R0480; RRID: AB_579538	1:500 (FC)
Mouse anti-human NTPDase3	www.ectonucleotidases-ab.com	Cat#hN3-B3S	5 μg/mL (FC)
ATF3	Santa Cruz	Cat#SC-188	1:1000 (WB)
BIP	Cell signaling	Cat##3177	1:1000 (WB)
MHC class I antibody (W6/32)	Enzo	Cat##ALX-805-711-C100	1:500 (FC)<br>1:1000 (ICC)
Insulin	DAKO	Cat#A0546	1:1000 (ICC)

Supplementary Methods

Culture of human EndoC-βH1 cells and human islets, and cell treatments

EndoC-βH1 cells were cultured in DMEM containing 5.6 mmol/l glucose (Gibco, Thermo-Fisher Scientific, Paisley, UK), 2% BSA fraction V, fatty acid free (Roche, Manheim, Germany), 50 µmol/l 2-mercaptoethanol (Sigma-Aldrich, Poole, UK), 10 mmol/l nicotinamide (Calbiochem, Darmstadt, Germany), 5.5 µg/ml transferrin (Sigma-Aldrich), 6.7 ng/ml selenite (Sigma-Aldrich), 100 units/ml penicillin and 100 µg/ml streptomycin (Lonza, Leusden, Netherlands) in Matrigel-fibronectin-coated plates[12].

After isolation, the human islets were cultured in M199 culture medium (5.5 mmol/l of glucose) before being sent to Brussels, Belgium, where they were dispersed and, in some experiments FACS-sorted for mRNA determination. Dispersed cells were cultured in Ham's F-10 medium containing 6.1 mM glucose (Gibco, Thermo-Fisher Scientific), 10% fetal bovine serum (FBS) (Gibco, Thermo-Fisher Scientific), 2 mM GlutaMAX (Sigma-Aldrich), 50 mM 3-isobutyl-1-methylxanthine (Sigma-Aldric), 1% BSA fraction V, fatty acid free (Roche), 50 U/ml penicillin and 50 mg/ml streptomycin (Lonza)[12]. All the results from experiments shown with EndoC-βH1 cells or human islet cells refer to independent biological samples.

EndoC-βH1 cells or pancreatic islets were treated with human IFNα 2000 U/ml (equivalent to 11.1 pg/ml) (PeproTech Inc., Rocky Hill, NJ) alone, or in combination with human IL1β, 50 U/ml (equivalent to 240 pg/ml) (R&D Systems, Abingdon, UK), for the indicated time points, based on previous dose-response experiments[7]. Of note, peripheral blood levels of IFNα in individuals affected by T1D are in the range of 0 - 30 pg/ml and infection of peripheral blood mononuclear cells (PBMCs) from T1D patients with Coxsackievirus B4, a strain previously identified in the pancreas of T1D patients[13], produces IFNα concentration in the range of 0-65 pg/ml[14]. Patients with severe and complicated hepatitis C have serum IL1β concentrations in the range of 0.7 – 187 pg/ml[15]. The different time points were selected to represent early, intermediary and late responses to IFNα. In some conditions the cells were pre-treated for 2h with chemical inhibitors of JAK signaling (Baricitinib), bromodomain and extra-terminal proteins (BET) (JQ1+ and I-BET-151) (Selleckchem, Munich, Germany) or their respective vehicles. These compounds were maintained in the medium during the subsequent exposure to cytokines.

Tissue

Formalin-fixed paraffin embedded pancreatic sections from the Exeter Archival Diabetes Biobank (EADB; http://foulis.vub.ac.be/) or from the DiViD biopsy study of living donors with recent-onset T1D[16] were studied . These comprised 13 samples in total; 6 from non-diabetic subjects (age range: 4 - 58 years) and 7 from individuals with T1D (age range: 1.3 - 42 years; duration of disease range 3 days - 18 months; Supplementary Table 2). All samples were studied with full ethical approval and using adequate reporting standards for the study of human tissues (15/W/0258).

ATAC sequencing processing and analysis

Sequenced reads were mapped to the human reference genome version hg19 using bowtie2 (version 2.3.4.1)[17] and unaligned reads were filtered out. Afterwards, duplicate reads were removed using Picard MarkDuplicates (version 2.5.0) (Picard Tools, retrieved November 29, 2018, from https://broadinstitute.github.io/picard/index.html) and reads aligned to the mitochondrial DNA or to ENCODE blacklisted regions[18] were filtered out using samtools (version 1.8)[19]. Finally, ATAC-seq reads were shifted 4bp in the forward strand and -5bp on the reverse strand.

Bam files from different replicates were merged into a single one using samtools (version 1.8)[19] and peaks were called from that merged file using MACS2 callpeak (version 2.1)[20] using the parameters "-q 0.05 --nomodel --shift -100 --extsize 200".

Differential analysis of ATAC-seq chromatin accessibility was performed using the R package DESeq2 [21]. A consensus peak set was generated for each timepoint by merging the called peaks. Then, reads were counted at each peak and the resulting matrix was used as input for DESeq2. Briefly, DESeq2 normalizes samples according to per-sample sequencing depth and accounting for intra-sample variability. Then, it fits data to a negative binomial generalized linear model (GLM) and calculates the Wald statistic. Finally, raw p-values are false discovery rate (FDR)-corrected for multiple testing using the Benjamini-Hochberg method. Peaks with an absolute $\log_2$ fold change higher than 1 ($|\log_2 FC|>1$) and an FDR < 0.05 were considered differentially accessible after the IFNα treatment.

RNA sequencing processing and analysis

Sequenced reads were mapped to the human genome (version GRCh37/hg19) using the software Tophat 2 (v2.0.13)[22] with default parameters. Mapped reads were then annotated based on the Gencode version 18[23] using Flux Capacitor[24] (http://confluence.sammeth.net) with default parameters. The relative expression of genes and transcripts is represented in RPKM units (''reads per kilobase per million mapped reads'')[25].

The genes and transcripts differentially expressed after IFNα exposure were identified by using the R/Bioconductor package EdgeR[26]. For the present analysis read counts were normalized using the Trimmed Mean of M-values (TMM) method, which calculates a set of normalization factors, one for each sample, and try to eliminate composition biases between libraries. The dispersion estimates was obtained and the negative binomial generalized linear model fitted. Different contrasts were used to allow the evaluation among the time points. The differential expression was determined using the quasi-likelihood (QL) F-test. Genes or transcripts were considered differentially expressed if presented a FDR < 0.05 and a $|\log_2 FC| > 0.58$ (FC > 1.5).

Alternative splicing (AS) events were analyzed by rMATS[27] using paired settings. This generated percentage splicing index (PSI) values and the false discovery rates for five different splicing events:

cassette exons (CEx), mutually exclusive exons (MXE), retained introns (RI), and 5' and 3' alternative splice sites (5/3ASS). To select the more biologically relevant AS events a threshold of $|\Delta PSI| > 20\%$ and FDR < 0.05% was applied. The computational motif enrichment analysis of RNA-binding proteins (RBPs) in the vicinity of alternatively spliced cassette exons was performed using rMAPS[28] by comparing the spatial occurrence of RBPs motifs among flanking regions of exons whose inclusion/exclusion was modified by IFNα-exposure to the one non-modified. Functional impact of the splicing changes was performed using the Exon Ontology database[29].

The quantification of alternative first exon (AFE) usage from RNA-seq data was performed using the software SEASTAR[30]. In brief, all the putative non-redundant first exons (FEs) were identified by transcript assembly from our RNA-sequencing and then their usage was quantified by counting the reads aligned to the FEs and to their downstream splice junctions. Finally, the PSI values for AFEs detected in each sample were calculated and the rMATS statistical method[27] used to determine whether there was significant differential usage induced by IFNα. The standard parameters were used, except: '-c 0.05 -t P'.

RNA polymerase II (POLR2A) ChIP-seq of human K562 cells exposed to IFNα for 6h was obtained from the ENCODE project (GSM935474); signal tracks represent the control-normalized tag density (bigWig format) of pooled replicates. The signal is expressed as p-value to reject the null hypothesis that the signal at that location is present in the control.

Sequence conservation analysis

The sequence conservation among stable and gained open chromatin regions detected by ATAC-seq was assessed by determining average phastCons 46 way score in placental mammals[31]. The scores average was calculated in 50bp bins over a 2kb window center on the open chromatin site. Such regions were then randomized along the mappable genome using regioneR[32] and used as control.

Assignment of open chromatin regions to target genes

To annotate open chromatin as distal or proximal, we assigned each ATAC-seq region to the nearest major TSS of a coding gene (the most upstream annotated TSS) using information from Gencode version 18[23]. Regions closer than 2kb to the nearest TSS were annotated as promoters, all the other regions were considered as distal regulatory elements.

We evaluated the correlation between different classes of open chromatin and changes in mRNA expression and protein abundance (Figs. 1C-F, Supplementary Fig. 3E), and assigned ATAC-seq regions to a gene when closer than 20kb of its TSS.

Annotation of CAGE-defined TSSs

The human TSSs peaks detected by genome-wide 5'-RNA sequencing of capped RNAs (Cap Analysis of Gene Expression, CAGE) were obtained from the FANTOM5 database[33] (http://fantom.gsc.riken.jp/5/datafiles/phase2.5/extra/CAGE_peaks/). Canonical TSSs were defined as

the most upstream annotated TSS based on the Gencode version 18. All the other TSSs non-overlapping the principal TSS and Gencode-annotated were defined as alternative TSSs. Only TSSs having peaks with > 10 Tags Per Million (TPM) were considered in the analysis.

The promoter regions were considered as $\pm$ 2kb around the TSS peak, and defined based on the type of TSS (canonical or alternative). The overlap between OCRs and promoters was performed using the function *overlapRegions* from the R package regioneR[32], with standard parameters.

For visualization, CAGE tag start site (CTSS) human tracks from the FANTOM5 Phase1+2[33] were pooled and filtered with 3 or more tags per library. After that, RLE (Relative Log Expression)-factor normalization was applied and the results are represented using a log scale.

Proteomics processing

Protein extracts were dissolved in 50 mM NH_4HCO_3 containing 8 M urea and 10 mM dithiothreitol and vortexed at 800 rpm for 1h at 37ºC. Sulfydryl groups were alkylated by using 400 mM iodoacetamide (40 mM final concentration), and incubating for 1h in the dark at room temperature. Samples were then diluted 8-fold with 50 mM NH_4HCO_3, and $CaCl_2$ was added to a final concentration of 1 mM. The digestion was performed with trypsin at 1:50 enzyme:protein ratio and incubation at 37ºC for 5h. The final peptides were extracted using C18 cartridges (Discovery, 50 mg, Sulpelco) and concentrated in a vacuum centrifuge. Peptides quantification was done by BCA, normalized and labeled with tandem mass tags (TMT-10plex, ThermoFisher Scientific) according to the manufacturer's instructions. Labeled peptides were extracted using C18 cartridges and fractionated into 24 fractions using high-pH reversed phase chromatography. Peptide fractions were loaded into a C18 column (70 cm × 75 µm i.d. packed with Phenomenex Jupiter, 3 µm particle size, 300 Å pore size) connected to Waters NanoAquity UPLC system. A gradient of water (solvent A) and acetonitrile (solvent B), both containing 0.1% formic acid, was used to elute the peptides, which were directly analyzed by nanoelectrospray ionization on a Q-Exactive mass spectrometer (Thermo Fisher Scientific). Scans were collected with a resolution of 35,000 at 400 m/z in a 400-2000 m/z range. High-energy collision induced dissociation (HCD) fragmentation were set for the 12 most intense parent ions using the following parameters: peptide charge $\geq$ 2, 2.0 m/z isolation width, 30% normalized collision energy and 17,500 resolution at 400 m/z. Each parent ion was fragmented only once before being dynamically excluded for 30s.

Collected data were processed using Decon2LS_v2.0 [34] and DTARefinery[35], both with default parameters, to generate peak lists. Peptide identification was done using MS-GF+[36] by searching peak lists against islet protein sequences deduced from transcriptomic experiments[37] and supplemented with keratin sequences (32,780 total protein sequences). For MS-GF+ searches, a parent ion mass tolerance of 10 ppm, partially tryptic digestion and 2 missed cleavages were allowed. The following modifications were also considered during searches: cysteine carbamidomethylation and N-terminal/lysine TMT

addition as static modifications, and methionine oxidation as a variable modification. Results were filtered in two steps to a final false-discovery rate <1%: spectral-peptide matches - MS-GF probability < 10^{-9}, and protein level < 10^{-10}. The intensity of TMT reporter ions was extracted using MASIC[38]. Finally, the data was $\log_2$ converted and normalized by standard median centering. Proteins were quantified using a Bayesian proteoform discovery methodology (BP-Quant) in combination with standard reference-based median quantification[39]. Proteins were considered significant with a cutoff of $p \leq 0.05$ based on a paired *t*-test.

Dynamic Regulatory Events Miner (DREM) modeling

The DREM divided genes in paths based on their expression profiles and identified bifurcation points, which are moments in time series where previously co-expressed groups of genes start to diverge. These points were then annotated with the TFs potentially regulating the split. The following parameters were used: Minimum Absolute Expression Change = 1.5 (difference from 0); Model Selection Options = Penalized Likelihood, Node Penalty: 40; Expression Scaling Options = Incorporate expression in regulator data for TF; default values were applied for other parameters. The key TFs were selected based on the split scores (score threshold < 0.05).

In order to validate the binding of these key TFs in promotor regions, but also in distant REs, we have integrated the DREM outputs with the changes in chromatin accessibility induced by IFNα. For this purpose, we first associated each open chromatin peak to the nearest TSS of a gene (if closer than 1Mb) and assigned the DREM pathway of the gene to the associated ATAC-seq peak. Next, binding motif matrices from TF appearing in the DREM pathways were selected from JASPAR2016 R package[40]. Footprint was assayed for each relevant TF in ATAC-seq peaks annotated to its DREM pathway by using factorFootprints function from the R package ATACseqQC[41] specifying a minimum score of 95% for finding a motif match. The genes selected for validation with specific siRNAs were identified based on the presence of predicted binding sites for the targeted TF using the i-cisTarget tool[42].

Functional enrichment evaluation

The functional enrichment results were generated using the Gene Set Enrichment Analysis (GSEA) software v3.0[43] when comparing full list of genes (Supplementary Figs. 4A and B). For the functional analysis of WGCNA modules (Fig. 3G) and T1D risk genes (Supplementary Figs. 2A and B) the hypergeometric distribution was used to estimate the significance of enriched pathways and biological processes using g:Profiler[44]. The whole list of genes identified by RNA-seq with a mean RPKM > 0.5 in at least one condition was used as background. The standard parameters were applied in both methods, except for minimum and maximum size of functional category values that were adjusted to 5 and 350, respectively. Enrichment maps of significantly modified biological process (Gene Ontology (GO)) were

generated using the plugin "Enrichment Map" v3.1[45] and visualized within Cytoscape v3.6, using standard parameters.

T1D risk genes were identified from immunobase (www.immunobase.org, accessed on 11/2018), GWAS catalog (https://www.ebi.ac.uk/gwas/, accessed on 11/2018) and[46]. The risk genes were selected based on the following criteria: 1) T1D as the Disease/Trait evaluated by the study, 2) a p-value for the lead SNP < 0.5×10^{-8}, 3) selecting the reported genes linked to the lead SNP by the original study, 4) filtering only the reported genes expressed in human islets (mean RPKM > 0.5 at basal condition or after exposure to IL1β+IFNγ)[2,37] for functional enrichment analysis as described above.

Real-time PCR

The real-time PCR quantification was performed using SYBR Green. Gene expression values were normalized by the housekeeping gene β-actin, as its expression is not affected by the present experimental conditions[7], and then by the highest value of each experiment considered as 1. The sequences of primers used are shown in Supplementary Table 3.

Western blot, immunocytochemistry, ELISA and flow cytometry analysis

For Western blot analysis, the cells were lysed using Laemmli buffer. Total protein was resolved by 10% SDS-PAGE gel and then transferred to nitrocellulose membranes. Immunoblot analysis was performed with specific antibodies as indicated in Supplementary Table 5. Peroxidase-conjugated antibodies were used as secondary antibodies (Supplementary Table 5). The detection of immunoreactive bands was performed using chemiluminescent substrate (SuperSignal West Femto, Thermo Scientific, Chicago, USA) in a Bio-Rad chemi DocTM XRS+ system (Bio-Rad laboratories). The ImageLab software (Bio-Rad laboratories) was used for densitometric quantification of the bands. The values obtained were normalized by the housekeeping protein α-tubulin and then by the highest value of each experiment considered as 1.

Cell supernatants of EndoC-βH1 cells exposed or not to IFNα for 24h were retrieved and human HLA-E protein expression was measured by enzyme-linked immunosorbent assay (Biorbyt, Cambridge, UK).

For the flow cytometry analysis of HLA-E and MHC class I the EndoC-βH1 cells were seeded in 24-well plates (300,000 cells per condition) 72h before being exposed or not to IFNα for 24h. Next, the cells were incubated with primary antibody (mouse monoclonal anti-HLA-E antibody clone 3D12 (1:250) (Biolegend, San Diego, USA) or rabbit anti-MHC class I (W6/32) (Enzo Life Sciences, NY, USA) (1:1000)) for 2h at 4°C, without permeabilization, and subsequently with secondary antibody conjugated with fluorescent dyes for 1h at 4°C (1:500) (Alexa Fluor™ anti-mouse or anti-rabbit) before performing flow cytometry analysis (BD LSRFortessa™ X-20, San Jose, CA, USA). Data analysis and graphical representation were performed using FlowJo software version v10 (Tree Star, Ashland, USA). The gating strategy is represented in Supplementary Fig. 5L. In summary, the cells were identified by FSC/SSC

morphological gates (to exclude debris and dead cells). Next, the FSC-W and FSC-H relation was used to eliminate doublets of cells. Finally, histogram distributions of the fluorophore were generated and the mean fluorescence intensity determined. The positive and negative thresholds were set using an isotype Ig control with the same fluorophore. For immunocytochemistry (ICC), 30,000 cells per condition were plated on polylysine-coated coverslips, then treated with IFNα for 24h and fixed with 4% paraformaldehyde. Cells were permeabilized with 0.1 % Triton X100 for 5 min and after incubated with rabbit anti-MHC class I (W6/32) (1:1000) and mouse monoclonal anti-insulin (1:1000). Alexa Fluor-conjugated secondary antibodies were used (Supplementary Table 5). After nuclear staining with Hoechst 33342 (HO), coverslips were mounted with fluorescent mounting medium (Dako, Carpintera, CA, USA) and immunofluorescence was visualized on a Zeiss microscope (Zeiss-Vision, Munich, Germany). Images were obtained at ×20 or ×40 magnification and analysed using AxioVision software (version 4.7.2; Zeiss-Vision, Munich, Germany).

Small-RNA interference

For transfection, the siRNA and the Lipofectamine RNAiMAX were diluted separately in OptiMEM medium and incubated at room temperature for 5 min. The Lipid-siRNA complexes were then formed at room temperature for 20 min in a proportion of 1.3 µl Lipofectamine RNAiMAX to 150 nmol/l of siRNA. The complexes were diluted five times in antibiotic-free medium and added to the cells at a final concentration of 30 nmol/l siRNA for overnight transfection.

The non-specific control siRNA (siCT) (Allstar Negative Control siRNA (Qiagen, Netherlands) does not affect gene expression or insulin secretion by human beta cells[47]. The concentration of siRNAs presently used (30 nM) was selected based on dose-response studies[47].

Cell viability assessment

The percentage of viable, apoptotic and necrotic cells was determined using nuclear dyes (propidium iodide (10 µg/ml, Sigma) and Hoechst 33342 (10 µg/ml, Sigma)). This method has been validated for use in pancreatic beta cells by systematic comparison with electron microscopy, caspase 3 activation and DNA laddering[48-50]. A minimum of 500 cells was counted per condition. Viability was evaluated by two independent observers, one of them being unaware of sample identity. The agreement between the two observers was > 90%.

Supplementary References:

1 Ernst, J., Vainas, O., Harbison, C. T., Simon, I. & Bar-Joseph, Z. Reconstructing dynamic regulatory maps. *Mol Syst Biol* **3**, 74, doi:10.1038/msb4100115 (2007).

2 Gonzalez-Duque, S. *et al.* Conventional and Neo-antigenic Peptides Presented by beta Cells Are Targeted by Circulating Naive CD8+ T Cells in Type 1 Diabetic and Healthy Donors. *Cell Metab* **28**, 946-960 e946, doi:10.1016/j.cmet.2018.07.007 (2018).

3 Russell, M. A. *et al.* HLA Class II Antigen Processing and Presentation Pathway Components Demonstrated by Transcriptome and Protein Analyses of Islet beta-Cells From Donors With Type 1 Diabetes. *Diabetes* **68**, 988-1001, doi:10.2337/db18-0686 (2019).

4 Xin, Y. *et al.* RNA Sequencing of Single Human Islet Cells Reveals Type 2 Diabetes Genes. *Cell Metab* **24**, 608-615, doi:10.1016/j.cmet.2016.08.018 (2016).

5 Heinz, S. *et al.* Simple combinations of lineage-determining transcription factors prime cis-regulatory elements required for macrophage and B cell identities. *Mol Cell* **38**, 576-589, doi:10.1016/j.molcel.2010.05.004 (2010).

6 Colli, M. L. *et al.* PDL1 is expressed in the islets of people with type 1 diabetes and is up-regulated by interferons-alpha and-gamma via IRF1 induction. *EBioMedicine* **36**, 367-375, doi:10.1016/j.ebiom.2018.09.040 (2018).

7 Marroqui, L. *et al.* Interferon-alpha mediates human beta cell HLA class I overexpression, endoplasmic reticulum stress and apoptosis, three hallmarks of early human type 1 diabetes. *Diabetologia* **60**, 656-667, doi:10.1007/s00125-016-4201-3 (2017).

8 Langfelder, P., Luo, R., Oldham, M. C. & Horvath, S. Is my network module preserved and reproducible? *PLoS Comput Biol* **7**, e1001057, doi:10.1371/journal.pcbi.1001057 (2011).

9 Li, T. *et al.* A scored human protein-protein interaction network to catalyze genomic interpretation. *Nat Methods* **14**, 61-64, doi:10.1038/nmeth.4083 (2017).

10 Subramanian, A. *et al.* A Next Generation Connectivity Map: L1000 Platform and the First 1,000,000 Profiles. *Cell* **171**, 1437-1452 e1417, doi:10.1016/j.cell.2017.10.049 (2017).

11 Pasciuto, E. & Bagni, C. SnapShot: FMRP mRNA targets and diseases. *Cell* **158**, 1446-1446 e1441, doi:10.1016/j.cell.2014.08.035 (2014).

12 Brozzi, F. *et al.* Cytokines induce endoplasmic reticulum stress in human, rat and mouse beta cells via different mechanisms. *Diabetologia* **58**, 2307-2316, doi:10.1007/s00125-015-3669-6 (2015).

13 Dotta, F. *et al.* Coxsackie B4 virus infection of beta cells and natural killer cell insulitis in recent-onset type 1 diabetic patients. *Proc Natl Acad Sci U S A* **104**, 5115-5120, doi:10.1073/pnas.0700442104 (2007).

14 Xia, C. Q. *et al.* Increased IFN-alpha-producing plasmacytoid dendritic cells (pDCs) in human Th1-mediated type 1 diabetes: pDCs augment Th1 responses through IFN-alpha production. *J Immunol* **193**, 1024-1034, doi:10.4049/jimmunol.1303230 (2014).

15 Antonelli, A. *et al.* Serum levels of proinflammatory cytokines interleukin-1beta, interleukin-6, and tumor necrosis factor alpha in mixed cryoglobulinemia. *Arthritis Rheum* **60**, 3841-3847, doi:10.1002/art.25003 (2009).

16 Krogvold, L. *et al.* Pancreatic biopsy by minimal tail resection in live adult patients at the onset of type 1 diabetes: experiences from the DiViD study. *Diabetologia* **57**, 841-843, doi:10.1007/s00125-013-3155-y (2014).

17 Langmead, B. & Salzberg, S. L. Fast gapped-read alignment with Bowtie 2. *Nat Methods* **9**, 357-359, doi:10.1038/nmeth.1923 (2012).

18 Consortium, E. P. An integrated encyclopedia of DNA elements in the human genome. *Nature* **489**, 57-74, doi:10.1038/nature11247 (2012).

19 Li, H. *et al.* The Sequence Alignment/Map format and SAMtools. *Bioinformatics* **25**, 2078-2079, doi:10.1093/bioinformatics/btp352 (2009).

20 Zhang, Y. *et al.* Model-based analysis of ChIP-Seq (MACS). *Genome Biol* **9**, R137, doi:10.1186/gb-2008-9-9-r137 (2008).
21 Love, M. I., Huber, W. & Anders, S. Moderated estimation of fold change and dispersion for RNA-seq data with DESeq2. *Genome Biol* **15**, 550, doi:10.1186/s13059-014-0550-8 (2014).
22 Kim, D. *et al.* TopHat2: accurate alignment of transcriptomes in the presence of insertions, deletions and gene fusions. *Genome Biol* **14**, R36, doi:10.1186/gb-2013-14-4-r36 (2013).
23 Frankish, A. *et al.* GENCODE reference annotation for the human and mouse genomes. *Nucleic Acids Res* **47**, D766-D773, doi:10.1093/nar/gky955 (2019).
24 Montgomery, S. B. *et al.* Transcriptome genetics using second generation sequencing in a Caucasian population. *Nature* **464**, 773-777, doi:10.1038/nature08903 (2010).
25 Mortazavi, A., Williams, B. A., McCue, K., Schaeffer, L. & Wold, B. Mapping and quantifying mammalian transcriptomes by RNA-Seq. *Nat Methods* **5**, 621-628, doi:10.1038/nmeth.1226 (2008).
26 Robinson, M. D., McCarthy, D. J. & Smyth, G. K. edgeR: a Bioconductor package for differential expression analysis of digital gene expression data. *Bioinformatics* **26**, 139-140, doi:10.1093/bioinformatics/btp616 (2010).
27 Shen, S. *et al.* rMATS: robust and flexible detection of differential alternative splicing from replicate RNA-Seq data. *Proc Natl Acad Sci U S A* **111**, E5593-5601, doi:10.1073/pnas.1419161111 (2014).
28 Park, J. W., Jung, S., Rouchka, E. C., Tseng, Y. T. & Xing, Y. rMAPS: RNA map analysis and plotting server for alternative exon regulation. *Nucleic Acids Res* **44**, W333-338, doi:10.1093/nar/gkw410 (2016).
29 Tranchevent, L. C. *et al.* Identification of protein features encoded by alternative exons using Exon Ontology. *Genome Res* **27**, 1087-1097, doi:10.1101/gr.212696.116 (2017).
30 Qin, Z., Stoilov, P., Zhang, X. & Xing, Y. SEASTAR: systematic evaluation of alternative transcription start sites in RNA. *Nucleic Acids Res* **46**, e45, doi:10.1093/nar/gky053 (2018).
31 Siepel, A. *et al.* Evolutionarily conserved elements in vertebrate, insect, worm, and yeast genomes. *Genome Res* **15**, 1034-1050, doi:10.1101/gr.3715005 (2005).
32 Gel, B. *et al.* regioneR: an R/Bioconductor package for the association analysis of genomic regions based on permutation tests. *Bioinformatics* **32**, 289-291, doi:10.1093/bioinformatics/btv562 (2016).
33 Consortium, F. *et al.* A promoter-level mammalian expression atlas. *Nature* **507**, 462-470, doi:10.1038/nature13182 (2014).
34 Mayampurath, A. M. *et al.* DeconMSn: a software tool for accurate parent ion monoisotopic mass determination for tandem mass spectra. *Bioinformatics* **24**, 1021-1023, doi:10.1093/bioinformatics/btn063 (2008).
35 Petyuk, V. A. *et al.* DtaRefinery, a software tool for elimination of systematic errors from parent ion mass measurements in tandem mass spectra data sets. *Mol Cell Proteomics* **9**, 486-496, doi:10.1074/mcp.M900217-MCP200 (2010).
36 Kim, S. & Pevzner, P. A. MS-GF+ makes progress towards a universal database search tool for proteomics. *Nat Commun* **5**, 5277, doi:10.1038/ncomms6277 (2014).
37 Eizirik, D. L. *et al.* The human pancreatic islet transcriptome: expression of candidate genes for type 1 diabetes and the impact of pro-inflammatory cytokines. *PLoS Genet* **8**, e1002552, doi:10.1371/journal.pgen.1002552 (2012).
38 Monroe, M. E., Shaw, J. L., Daly, D. S., Adkins, J. N. & Smith, R. D. MASIC: a software program for fast quantitation and flexible visualization of chromatographic profiles from detected LC-MS(/MS) features. *Comput Biol Chem* **32**, 215-217, doi:10.1016/j.compbiolchem.2008.02.006 (2008).
39 Webb-Robertson, B. J. *et al.* Bayesian proteoform modeling improves protein quantification of global proteomic measurements. *Mol Cell Proteomics* **13**, 3639-3646, doi:10.1074/mcp.M113.030932 (2014).

40 Tan, G. JASPAR2016: Data package for JASPAR 2016. R package version 1.12.0, http://jaspar.genereg.net/. (2019).

41 Ou, J. *et al.* ATACseqQC: a Bioconductor package for post-alignment quality assessment of ATAC-seq data. *BMC Genomics* **19**, 169, doi:10.1186/s12864-018-4559-3 (2018).

42 Imrichova, H., Hulselmans, G., Atak, Z. K., Potier, D. & Aerts, S. i-cisTarget 2015 update: generalized cis-regulatory enrichment analysis in human, mouse and fly. *Nucleic Acids Res* **43**, W57-64, doi:10.1093/nar/gkv395 (2015).

43 Subramanian, A. *et al.* Gene set enrichment analysis: a knowledge-based approach for interpreting genome-wide expression profiles. *Proc Natl Acad Sci U S A* **102**, 15545-15550, doi:10.1073/pnas.0506580102 (2005).

44 Reimand, J. *et al.* g:Profiler-a web server for functional interpretation of gene lists (2016 update). *Nucleic Acids Res* **44**, W83-89, doi:10.1093/nar/gkw199 (2016).

45 Merico, D., Isserlin, R., Stueker, O., Emili, A. & Bader, G. D. Enrichment map: a network-based method for gene-set enrichment visualization and interpretation. *PLoS One* **5**, e13984, doi:10.1371/journal.pone.0013984 (2010).

46 Cooper, N. J. *et al.* Type 1 diabetes genome-wide association analysis with imputation identifies five new risk regions. *bioRxiv*, doi:10.1101/120022 (2017).

47 Moore, F., Cunha, D. A., Mulder, H. & Eizirik, D. L. Use of RNA interference to investigate cytokine signal transduction in pancreatic beta cells. *Methods Mol Biol* **820**, 179-194, doi:10.1007/978-1-61779-439-1_11 (2012).

48 Moore, F. *et al.* PTPN2, a candidate gene for type 1 diabetes, modulates interferon-gamma-induced pancreatic beta-cell apoptosis. *Diabetes* **58**, 1283-1291, doi:10.2337/db08-1510 (2009).

49 Rasschaert, J. *et al.* Toll-like receptor 3 and STAT-1 contribute to double-stranded RNA+ interferon-gamma-induced apoptosis in primary pancreatic beta-cells. *J Biol Chem* **280**, 33984-33991, doi:10.1074/jbc.M502213200 (2005).

50 Cunha, D. A. *et al.* Initiation and execution of lipotoxic ER stress in pancreatic beta-cells. *J Cell Sci* **121**, 2308-2318, doi:10.1242/jcs.026062 (2008).
