# Supplementary material for: An integrated multi-omics approach identifies the landscape of interferon-α-mediated responses of human pancreatic beta cells
Source: Nat Commun. 2020 May 22;11:2584. doi: 10.1038/s41467-020-16327-0 (PMC7244579; doi:10.1038/s41467-020-16327-0)
Supplement: Supplementary file 3 — Description of Additional Supplementary Files [file 41467_2020_16327_MOESM3_ESM.pdf]

Description of Additional Supplementary Files

File Name: Supplementary Data 1
Description: Lists of genes used in the RRHO comparisons

File Name: Supplementary Data 2
Description: List of the open chromatin regions (OCRs) at 2 and 24h, their fold changes, their classification and the annotation of the TSS of protein-coding genes located < 20Kb from the OCR

File Name: Supplementary Data 3
Description: List of differentially expressed genes from RNA-seq of EndoC-BH1 cells exposed to IFNa for the indicated time points

File Name: Supplementary Data 4
Description: List of differentially abundant proteins from proteomics of EndoC-BH1 cells exposed to IFNa for the indicated time points

File Name: Supplementary Data 5
Description: List of differentially expressed genes from RNA-seq of pancreatic human islets exposed to IFNa for the indicated time points

File Name: Supplementary Data 6
Description: List of differentially expressed transcripts from RNA-seq of EndoC-BH1 cells exposed to IFNa for the indicated time points

File Name: Supplementary Data 7
Description: List of differentially expressed transcripts from RNA-seq of pancreatic human islets exposed to IFNa for the indicated time points
