# Supplementary material for: An integrated multi-omics approach identifies the landscape of interferon-α-mediated responses of human pancreatic beta cells
Source: Nat Commun. 2020 May 22;11:2584. doi: 10.1038/s41467-020-16327-0 (PMC7244579; doi:10.1038/s41467-020-16327-0)
Supplement: Supplementary file 11 — Reporting Summary [file 41467_2020_16327_MOESM11_ESM.pdf]

nature research

Corresponding author(s): Maikel Luis Colli

Last updated by author(s): Apr 15, 2020

Reporting Summary

Nature Research wishes to improve the reproducibility of the work that we publish. This form provides structure for consistency and transparency in reporting. For further information on Nature Research policies, see Authors & Referees and the Editorial Policy Checklist.

Statistics

For all statistical analyses, confirm that the following items are present in the figure legend, table legend, main text, or Methods section.

n/a	Confirmed	
☐	☒	The exact sample size (*n*) for each experimental group/condition, given as a discrete number and unit of measurement
☐	☒	A statement on whether measurements were taken from distinct samples or whether the same sample was measured repeatedly
☐	☒	The statistical test(s) used AND whether they are one- or two-sided<br>*Only common tests should be described solely by name; describe more complex techniques in the Methods section.*
☒	☐	A description of all covariates tested
☒	☐	A description of any assumptions or corrections, such as tests of normality and adjustment for multiple comparisons
☐	☒	A full description of the statistical parameters including central tendency (e.g. means) or other basic estimates (e.g. regression coefficient) AND variation (e.g. standard deviation) or associated estimates of uncertainty (e.g. confidence intervals)
☐	☒	For null hypothesis testing, the test statistic (e.g. *F*, *t*, *r*) with confidence intervals, effect sizes, degrees of freedom and *P* value noted<br>*Give P values as exact values whenever suitable.*
☒	☐	For Bayesian analysis, information on the choice of priors and Markov chain Monte Carlo settings
☐	☒	For hierarchical and complex designs, identification of the appropriate level for tests and full reporting of outcomes
☐	☒	Estimates of effect sizes (e.g. Cohen's *d*, Pearson's *r*), indicating how they were calculated

Our web collection on statistics for biologists contains articles on many of the points above.

Software and code

Policy information about availability of computer code

Data collection	Libraries were sequenced single-end (ATAC-seq) or paired-end (RNA-seq) on an Illumina HiSeq 2500.
Data analysis	ATAC-seq analysis: sequenced reads were mapped to the human reference genome version hg19 using bowtie2 (version 2.3.4.1), duplicate reads were removed using Picard Tools (version 2.5.0), reads aligned to the mitochondrial DNA or to ENCODE blacklisted regions 3 were filtered out using samtools (version 1.8), peaks were called from BAM files using MACS2 (version 2.1), differential analysis of ATAC-seq chromatin accessibility was performed using the R package DESeq2 (version 1.24.0); RNA-seq analysis: sequenced reads were mapped to the human reference genome version hg19 using Tophat 2 (version 2.0.13), mapped reads were annotated based on the Gencode version 18 using Flux Capacitor (version 1.2.4), genes and transcripts differentially expressed were identified by the R package EdgeR (version 3.26.0); Alternative splicing (AS) events were analyzed by rMATS (version 3.2.5). The quantification of alternative first exon (AFE) usage from RNA-seq data was performed using the software SEASTAR (version 1.0.1). Proteomics data were processed using Decon2LS (version 2.0 19) and DTARefinery (version 1.2.0.1). Peptide identification was done using MS-GF+ (version 2018.01.30). The intensity of TMT reporter ions was extracted using MASIC (version 2.8.6303). Gene expression profile with Dynamic Regulatory Events Miner (DREM) version 2.0.3, Flow cytometry analysis with FlowJo software (Version 10). Gene set enrichment analysis with GSEA software v3.0. Enrichment Maps were generated using Enrichment Map app version 3.1 and visualized using and visualized within Cytoscape v3.6. Clustering analysis of PPIs using the EAGLE algorithm with ClusterViz version 1.0.3. Western blot densitometry analysis with ImageLab software v3.0. Statistical analysis of confirmatory experiments using Prism GraphPad version 6.0. Immunocytochemistry images were captured using AxioVision software version 4.7.2 and processed using the Fiji software version 20191027-2045.

For manuscripts utilizing custom algorithms or software that are central to the research but not yet described in published literature, software must be made available to editors/reviewers. We strongly encourage code deposition in a community repository (e.g. GitHub). See the Nature Research guidelines for submitting code & software for further information.

Data

Policy information about availability of data

All manuscripts must include a data availability statement. This statement should provide the following information, where applicable:

- Accession codes, unique identifiers, or web links for publicly available datasets
- A list of figures that have associated raw data
- A description of any restrictions on data availability

All raw and processed ATAC and RNA sequencing data that support the findings of this study have been deposited in NCBI Gene Expression Omnibus (GEO) with the primary accession code GSE133221 (subseries are GSE133218: RNA-seq of EndoC-BH1 cells (https://www.ncbi.nlm.nih.gov/geo/query/acc.cgi?acc=GSE133218), GSE148058: RNA-seq of human islets (https://www.ncbi.nlm.nih.gov/geo/query/acc.cgi?acc=GSE148058), GSE133219: ATAC-seq of EndoC-BH1 cells (https://www.ncbi.nlm.nih.gov/geo/query/acc.cgi?acc=GSE133219)). The proteomics datasets have been submitted to Pride under identifier number PXD014244 (http://www.ebi.ac.uk/pride/archive/projects/PXD014244). The network of regulatory interactions can be obtained from RegNetwoks (http://www.regnetworkweb.org/download/RegulatoryDirections.zip). The DrugBank database v5.1 can be downloaded from (https://www.drugbank.ca/releases/5-1-0/downloads/all-full-database). The inBio Map protein-protein interaction (PPI) network database can be obtained from (https://www.intomics.com/inbio/api/data/map_public/2016_09_12/inBio_Map_core_2016_09_12.zip). The CAGE peaks from FANTOM5 database can obtained on (http://fantom.gsc.riken.jp/5/datafiles/phase2.5/extra/CAGE_peaks/). The Connectivity Map database can be accessed using the CLUE platform (https://clue.io). The RNA polymerase II (POLR2A) ChIP-seq of human K562 cells can be obtained from the ENCODE project (GSM935474, https://www.encodeproject.org/experiments/ENCSR000FAX/). The Exon Ontology database can be accessed from: http://fasterdb.ens-lyon.fr/ExonOntology/. The information about T1D risk genes can be found on immunobase (www.immunobase.org) and GWAS catalog (https://www.ebi.ac.uk/gwas/).

Field-specific reporting

Please select the one below that is the best fit for your research. If you are not sure, read the appropriate sections before making your selection.

☒ Life sciences ☐ Behavioural & social sciences ☐ Ecological, evolutionary & environmental sciences

For a reference copy of the document with all sections, see nature.com/documents/nr-reporting-summary-flat.pdf

Life sciences study design

All studies must disclose on these points even when the disclosure is negative.

Sample size	The sample size was estimated based on results obtained by testing different sample sizes and their power for the detection of differentially expressed genes as described in Schurch NJ et al (2016), RNA.
Data exclusions	No data were excluded from the analysis.
Replication	All the results from experiments shown with EndoC-βH1 cells or human islet cells refer to independent biological samples. All the attempts of replication were successful.
Randomization	No randomization was performed, samples were treated according to the same protocols side-by-side with the respective controls
Blinding	Cell viability was evaluated by two independent observers, one of them being unaware of sample identity. The agreement between the two observers was > 90%. For the other experiments the blinding was not relevant since they mostly involved complex multi-omics approaches. Furthermore, they were performed following pre-established protocols of treatment and each condition was processed in parallel, as described in a recent publication by our group (Ramos-Rodriguez M et al., Nature Genet, 51:1588-1595, 2019)

Reporting for specific materials, systems and methods

We require information from authors about some types of materials, experimental systems and methods used in many studies. Here, indicate whether each material, system or method listed is relevant to your study. If you are not sure if a list item applies to your research, read the appropriate section before selecting a response.

Materials & experimental systems

n/a	Involved in the study	
☐	☒	Antibodies
☐	☒	Eukaryotic cell lines
☒	☐	Palaeontology
☒	☐	Animals and other organisms
☐	☒	Human research participants
☒	☐	Clinical data

Methods

n/a	Involved in the study	
☒	☐	ChIP-seq
☐	☒	Flow cytometry
☒	☐	MRI-based neuroimaging

Antibodies

Antibodies used

Antibody Supplier Identifier Dilutiuon
HLA-E clone 3D12 (Flow cytometry (FC)) Biolegend Cat#342602; RRID: AB_1659247 1:250 (FC)
HLA-E clone MEM-E/02 (Western blot (WB) and IF) Abcam Cat#ab2216; RRID: AB_302895 1:500 (WB) 1:150 (IF)
IRF1 Cell signaling Cat#8478; RRID: AB_10949108 1:1000 (WB)
phospho-STAT1 Cell signaling Cat#9167; RRID: AB_561284 1:1000 (WB)
phospho-STAT2 Cell signaling Cat#88410; RRID:AB_2800123 1:1000 (WB)
total STAT1 Cell signaling Cat#14495; RRID: AB_2716280 1:1000 (WB)
total STAT2 Cell signaling Cat#72604; RRID:AB_2799824 1:1000 (WB)
a-tubulin Sigma Cat#T9026; RRID:AB_477593 1:5000 (WB)
Peroxidase- conjugated donkey anti-rabbit IgG Jackson ImmunoResearch Cat#715-036-152; RRID:AB_2340590 1:1000 (WB)
Peroxidase- conjugated donkey anti-mouse IgG Jackson ImmunoResearch Cat#711-036-150; RRID:AB_2340773 1:1000 (WB)
Alexa Fluor 488 goat anti-Guinea-Pig IgG Life technologies, USA Cat#A11073; RRID: AB_2534117 1:500 (ICC)
Alexa Fluor 568 rabbit anti-mouse IgG Life technologies, USA Cat#A11061; RRID: AB_2534108 1:500 (ICC)
Polyclonal Goat Anti-Mouse Immunoglobulins/RPE Goat F(ab')2 Dako Cat#R0480; RRID: AB_579538 1:500 (FC)
Mouse anti-human NTPDase3 www.ectonucleotidases-ab.com Cat#hN3-B3S 5 µg/mL (FC)
Insulin Dako Guinea-Pig Polyclonal Cat#A0546 1:1000 (ICC) 1:700 (IHC)
Glucagon Abcam Rabbit Monoclonal EP3070 Cat# 92517 1:4000 (IHC)
Somatostatin Abcam Rat Monoclonal M09204 Cat# 30788 1:200 (IHC)
ATF3 Santa Cruz Cat#SC-188 1:1000 (WB)
BIP Cell signaling Cat##3177 1:1000 (WB)
MHC class I antibody (W6/32) Enzo Cat##ALX-805-711-C100 1:500 (FC) 1:1000 (ICC)

Validation

-HLA-E clone 3D12 (Flow cytometry) Biolegend Cat#342602, this antibody has been validated by the supplier for flow cytometry and has reactivity against human HLA-E (https://www.biolegend.com/ja-jp/products/purified-anti-human-hla-e-antibody-5962)
-HLA-E clone MEM-E/02 (Western blot and IF) Abcam Cat#ab2216; this antibody has been validated by the supplier for Western blotting and has reactivity against human HLA-E (https://www.abcam.com/hla-e-antibody-mem-e02-ab2216.html)
-IRF1 Cell signaling Cat#8478;this antibody has been validated by the supplier for Western blotting and has reactivity against human, mouse and rat IRF1 (https://www.cellsignal.com/products/primary-antibodies/irf-1-d5e4-xp-rabbit-mab/8478)
-phospho-STAT1 Cell signaling Cat#9167; this antibody has been validated by the supplier for Western blotting and has reactivity against human and mouse phospho-STAT1 (https://www.cellsignal.com/products/primary-antibodies/phospho-stat1-tyr701-58d6-rabbit-mab/9167)
-phospho-STAT2 Cell signaling Cat#88410; this antibody has been validated by the supplier for Western blotting and has reactivity against human and rat phospho-STAT2 (https://www.cellsignal.com/products/primary-antibodies/phospho-stat2-tyr690-d3p2p-rabbit-mab/88410)
-total STAT1 Cell signaling Cat#14495; this antibody has been validated by the supplier for Western blotting and has reactivity against human, mouse and rat STAT1 (https://www.cellsignal.com/products/primary-antibodies/stat1-d4y6z-rabbit-mab/14995)
-total STAT2 Cell signaling Cat#72604; this antibody has been validated by the supplier for Western blotting and has reactivity against human and mouse STAT2 (https://www.cellsignal.com/products/primary-antibodies/stat2-d9j7l-rabbit-mab/72604)
-a-tubulin Sigma Cat#T9026;this antibody has been validated by the supplier for Western blotting and has reactivity against human, mouse, rat, chicken, bovine a-tubulin (https://www.sigmaaldrich.com/catalog/product/sigma/t9026)
-Peroxidase- conjugated donkey anti-rabbit IgG Jackson ImmunoResearch Cat#711-036-152; this antibody has been validated by the supplier for Western blotting and has reactivity against rabbit `Ab (https://www.jacksonimmuno.com/catalog/products/711-036-152)
-Peroxidase- conjugated donkey anti-mouse IgG Jackson ImmunoResearch Cat#715-036-150, this antibody has been validated by the supplier for Western blotting and has reactivity against mouse Ab (https://www.jacksonimmuno.com/catalog/products/715-036-150)
-mouse anti-human NTPDase3, hN3-B3S, this antibody has been validated by the supplier for Flow cytometry and has reactivity against human NTPDase3 (http://ectonucleotidases-ab.com/documents/janvier-2019/humanNTPDase3_mAb.pdf)
-Alexa Fluor 488 goat anti-Guinea-Pig IgG Life technologies, USA Cat#A11073, this antibody has been validated by the supplier for ICC (our application) and has reactivity against Guinea-Pig Ab (https://www.thermofisher.com/antibody/product/Goat-anti-Guinea-Pig-IgG-H-L-Highly-Cross-Adsorbed-Secondary-Antibody-Polyclonal/A-11073)
-Polyclonal Goat Anti-Mouse Immunoglobulins/RPE Goat F(ab')2 Dako Cat#R0480, this antibody has been validated by the supplier for Flow cytometry (our application) and has reactivity against mouse Ab (https://www.agilent.com/cs/library/packageinsert/public/104485002.PDF)
-Insulin Dako Guinea-Pig Polyclonal Cat#A0546, this antibody has been validated by the supplier for ICC and IHC (our application) and has reactivity against Human and Porcine Insulin (specificity 100%), (https://www.agilent.com/cs/library/packageinsert/public/307684EFG_02.pdf)
-Glucagon Abcam Rabbit Monoclonal EP3070, this antibody has been validated by the supplier for IHC (our application) and has reactivity against Human, mouse and rat Glucagon, (https://www.abcam.com/glucagon-antibody-ep3070-ab92517.html)
-Somatostatin Abcam Rat Monoclonal M09204, this antibody has been validated by the supplier for IHC (our application) and has reactivity against Human and mouse somatostatin (https://www.abcam.com/somatostatin-antibody-m09204-ab30788.html)
-ATF3 Santa Cruz Cat#SC-188, this antibody has been validated by the supplier for Western blotting (our application) and has reactivity against Human, mouse and rat ATF3, (https://www.scbt.com/p/atf-3-antibody-c-19, https://search.cosmobio.co.jp/cosmo_search_p/search_gate2/docs/SCB_/SC188.20070822.pdf)
-BIP Cell signaling Cat##3177, this antibody has been validated by the supplier for Western blotting (our application) and has reactivity against Human and mouse BIP, (https://www.cellsignal.com/products/primary-antibodies/bip-c50b12-rabbit-mab/3177),
-MHC class I antibody (W6/32) Enzo Cat##ALX-805-711-C100 1:1000, this antibody has been validated by the supplier for Flow

cytometry and ICC (our application) and has reactivity against Human, Bovine, Cat and Monkey, (https://www.enzolifesciences.com/ALX-805-711/mhc-class-i-monoclonal-antibody-w6-32/)

Eukaryotic cell lines

Policy information about cell lines

Cell line source(s)	The human pancreatic beta cell line EndoC-βH1 was kindly provided by Dr. R. Scharfmann, University of Paris, France
Authentication	EndoC-βH1 was authenticated by comparing its epigenome and transcriptome to that of human islets (which contain about 70% of β cells) and by testing their function (insulin release, expression of human beta cell markers).
Mycoplasma contamination	We confirm that cell line used was tested for Mycoplasm contamination and the results were negative.
Commonly misidentified lines (See ICLAC register)	No misidentified cell lines

Human research participants

Policy information about studies involving human research participants

Population characteristics	*Describe the covariate-relevant population characteristics of the human research participants (e.g. age, gender, genotypic information, past and current diagnosis and treatment categories). If you filled out the behavioural & social sciences study design questions and have nothing to add here, write "See above."*
Recruitment	*Describe how participants were recruited. Outline any potential self-selection bias or other biases that may be present and how these are likely to impact results.*
Ethics oversight	*Identify the organization(s) that approved the study protocol.*

Note that full information on the approval of the study protocol must also be provided in the manuscript.

Flow Cytometry

Plots

Confirm that:

☒ The axis labels state the marker and fluorochrome used (e.g. CD4-FITC).

☒ The axis scales are clearly visible. Include numbers along axes only for bottom left plot of group (a 'group' is an analysis of identical markers).

☒ All plots are contour plots with outliers or pseudocolor plots.

☒ A numerical value for number of cells or percentage (with statistics) is provided.

Methodology

Sample preparation	First, EndoC-βH1 cells were seeded in 24-well plates (300,000 cells per condition) 72h before being exposed or not to IFNα for 24h. Next, the cells were incubated with mouse monoclonal anti-HLA-E antibody clone 3D12 (1:250) (Biolegend, San Diego, USA) for 2h at 4oC, without permeabilization, and subsequently with secondary antibody conjugated with fluorescent dyes for 1h at 4oC (1:500) (Alexa Fluor™ anti-mouse 647) before performing flow cytometry analysis (BD LSRFortessa™ X-20, San Jose, CA, USA). Data analysis and graphical representation were performed using FlowJo software version v10 (Tree Star, Ashland, USA)
Instrument	BD LSRFortessa X-20
Software	FlowJo X
Cell population abundance	The samples analyzed by flow cytometry were from the same cell line (EndoC-βH1) exposed or not to IFNα. For each run, at least 30,000 cells were analyzed based on previous experience.
Gating strategy	Cell were identified by FSC/SSC morphological gates (to exclude debris) positive and negative threshold were set using an isotype Ig control with the same fluorophore.

☒ Tick this box to confirm that a figure exemplifying the gating strategy is provided in the Supplementary Information.
